# Muscle patterns underlying voluntary modulation of co-contraction

**DOI:** 10.1371/journal.pone.0205911

**Published:** 2018-10-19

**Authors:** Daniele Borzelli, Benedetta Cesqui, Denise J. Berger, Etienne Burdet, Andrea d’Avella

**Affiliations:** 1 Laboratory of Neuromotor Physiology, IRCCS Fondazione Santa Lucia, Rome, Italy; 2 Department of Biomedical and Dental Sciences and Morphofunctional Imaging, Università di Messina, Messina, Italy; 3 Centre of Space Bio-medicine, Università di Roma Tor Vergata, Rome, Italy; 4 Department of Bioengineering, Imperial College of Science, Technology and Medicine, London, United Kingdom; Universita degli Studi di Verona, ITALY

## Abstract

Manipulative actions involving unstable interactions with the environment require controlling mechanical impedance through muscle co-contraction. While much research has focused on how the central nervous system (CNS) selects the muscle patterns underlying a desired movement or end-point force, the coordination strategies used to achieve a desired end-point impedance have received considerably less attention. We recorded isometric forces at the hand and electromyographic (EMG) signals in subjects performing a reaching task with an external disturbance. In a virtual environment, subjects displaced a cursor by applying isometric forces and were instructed to reach targets in 20 spatial locations. The motion of the cursor was then perturbed by disturbances whose effects could be attenuated by increasing co-contraction. All subjects could voluntarily modulate co-contraction when disturbances of different magnitudes were applied. For most muscles, activation was modulated by target direction according to a cosine tuning function with an offset and an amplitude increasing with disturbance magnitude. Co-contraction was characterized by projecting the muscle activation vector onto the null space of the EMG-to-force mapping. Even in the baseline the magnitude of the null space projection was larger than the minimum magnitude required for non-negative muscle activations. Moreover, the increase in co-contraction was not obtained by scaling the baseline null space projection, scaling the difference between the null space projections in any block and the projection of the non-negative minimum-norm muscle vector, or scaling the difference between the null space projections in the perturbed blocks and the baseline null space projection. However, the null space projections in the perturbed blocks were obtained by linear combination of the baseline null space projection and the muscle activation used to increase co-contraction without generating any force. The failure of scaling rules in explaining voluntary modulation of arm co-contraction suggests that muscle pattern generation may be constrained by muscle synergies.

## Introduction

During daily life, we often perform manipulative actions that involve unstable interactions between the hand and the environment or the rejection of external disturbances, such as when working with tools. Successful performance of these actions requires controlling the endpoint impedance through muscle co-contraction [1,2,3,4]. Increasing endpoint impedance reduces the perturbing effect of an external force [2,3,5,6,7] and improves movement accuracy [8,9,10,11]. Impedance is modulated through muscle co-contraction to facilitate ball catching [12] while an overall decrease is associated with practice when learning a new motor task [13,14,15].

Arm impedance can be characterized by inertia, damping, and stiffness. Many studies have characterized these components both during static tasks [16,17,18,19,20,21,22,23] and dynamic tasks [3,6,7,8,11,24,25,26]. Because inertia cannot be controlled for a given arm posture, damping co-varies with stiffness, and mechanical interactions with the environment occur usually at the hand, past studies mostly focused on end-point stiffness for the characterization of the muscular control of impedance.

The musculoskeletal system can perform the same action with different end-point trajectories or force profiles, the same end-point trajectory or force profiles can be achieved with different joint trajectories or torque profiles, and the same joint trajectories or torque profiles can be generated by different muscle activation patterns [27]. Thus, infinitely many different muscle activation patterns generate the same action. While much research in motor control has focused on investigating how the CNS selects the muscle patterns underlying a desired movement or end-point force, the strategies used to coordinate many muscles to achieve a desired end-point impedance have received considerably less attention. Indeed, there is a redundancy resolution (and exploitation) problem also for the control of impedance. For an *antagonist* pair of muscles with opposite actions on a single joint, joint torque is the sum of the torques generated by each muscle, which have opposite signs. Thus, as each muscle torque is a function of muscle activation, net joint torque depends on the *difference* between the muscle activations appropriately weighted to account for the moment arms and forces of each muscle. In contrast, impedance depends on the *sum* of weighted muscle activations. One can then define the *co-contraction* as any activation of two muscles that generates no net torque (i.e. zero weighted activation difference) and the *level* of co-contraction as the specific value of the weighted sum of the two activations. However, for a multi-muscle multi-joint system such as the arm, there are infinite muscle co-contraction patterns that the CNS can select to achieve not only a desired end-point force but also a desired end-point impedance. How the CNS selects one of these infinite muscles patterns is still an open question.

Muscle activations underlying stiffness modulation have been studied during both pointing and force exertion tasks [28,29]. However, previous studies recorded only a few agonist-antagonist muscles pairs. A small number of muscles may not be adequate to characterize the coordination strategies that the CNS employs to exploit the flexibility deriving from the redundancy of the musculoskeletal system. Moreover, the identification of an agonist-antagonist muscle pair may be valid only for specific tasks and experimental constraints [30] and the notion of agonist-antagonist muscle pairs is not well defined for a multi-muscle multi-joint system. Thus, our goal was to characterize the muscle coordination strategies that the CNS uses to control impedance by recording from many arm muscles rather than a few agonist-antagonist pairs.

In a multi-muscle, multi-joint system, *co-contraction* requires a multivariate characterization. If we consider the vector space of all muscle activation patterns, co-contraction patterns constitute the null space of the mapping of muscle patterns onto end-point forces. If the mapping is linear, the null space is a vector subspace that can be directly characterized from the muscle-to-force matrix. Thus, if such matrix is known, it is possible to decompose muscle patterns into a force-generating component and a null space component. The CNS must change the null space component of the muscle activation vector to modulate impedance while maintaining a given end-point force. Thus, we designed an experimental protocol to investigate how the CNS changes muscle patterns and their null space projections during voluntary modulation of arm muscles co-contraction.

We used a multidirectional isometric force generation task with a simulated perturbation to investigate the components of the muscle activation vector in the null space. We asked participants to displace a cursor in a virtual reality environment by generating submaximal isometric forces to reach multiple targets in different spatial directions. We could then approximate the mapping of muscle activations (EMG) to end-point forces with a linear relation [29,31,32], estimate the EMG-to-force matrix by linear regression of the recorded forces and EMG, and compute the associated null space matrix. We then induced subjects to voluntary modulate co-contraction by simulating a disturbing force. We perturbed the cursor and we instructed subjects to reduce its oscillation by increasing the co-contraction of their arm muscles [33]. We used the projection of the instantaneous muscle activation vector in the null space to adjust the stiffness of the coupling between the hand and the cursor, thus allowing the subject to attenuate the effect of the perturbation by increasing co-contraction.

We first tested if subjects could succeed in this novel task by voluntarily increasing co-contraction to overcome the virtual disturbance. As all subjects could increase co-contraction by modulating the projection of the muscle activations in the null space, we then investigated how the CNS coordinates a redundant set of muscles to increase co-contraction. We characterized the modulation of the muscle activations both at the level of individual muscles and in terms of null space projections. We assessed how co-contraction affects the directional tuning of individual muscles by fitting cosine functions [32]. We then investigated how the null space projections of the muscle activation vectors change with different levels of co-contraction. We first tested if the CNS increases co-contraction by scaling of the null space vector observed during isometric force generation without any simulated instability (baseline condition). However, the null space projections of the muscle patterns in the baseline condition may derive from the skeletal geometry and the physiological non-negativity constraint for the muscle activations. In fact, even if the CNS selects muscle patterns by minimizing muscular effort, a non-zero null space component is required to achieve the minimum norm solution because of the non-negativity constraint. We then examined whether different co-contraction levels are obtained by affine scaling of the null space component that is added to the non-negative minimum norm muscle activation vector in the baseline condition. We also examined whether different co-contraction levels, recorded during perturbation conditions, are obtained by affine scaling of the null space component that is added to the muscle activation vector observed during the baseline condition in the perturbed conditions. Finally, we assessed whether the null space components of the perturbed conditions, in which both force and co-contraction are required, are obtained as a linear combination of the null space vector observed during the force only condition and the muscle activation vector observed during pure co-contraction condition.

## Materials and methods

Subjects were asked to apply isometric forces at the hand for displacing a cursor in a virtual reality environment, and to co-contract their arm muscles for attenuating the effect of a sinusoidal disturbance on the position of the cursor. The cursor’s movement was simulated as two connected mass-spring-damper systems. The position of the first mass was controlled by the isometric force applied by the subject. The position of the second mass, which was not affected by the position of the first mass, corresponded to the position of the cursor and was perturbed by a virtual sinusoidal force. The stiffness of the virtual spring connecting the two masses was adjusted in real-time by the projection of the muscle activation vector in the null space thus allowing the subject to attenuate the effect of the perturbation by increasing co-contraction. Therefore, the stiffness of the virtual spring during isometric force generation simulated the stiffness of the hand during a disturbance rejection task.

### Participants

Nine right handed subjects (age 23.8 ± 3.5, mean ± SD, 6 females) participated in the experiment after giving written informed consent. All procedures were conducted in conformity with the Declaration of Helsinki and were approved by the Ethical Review Board of the Santa Lucia Foundation (Prot. CE/AG4-PROG.222-34).

### Experimental apparatus and data acquisition

**Fig 1:**
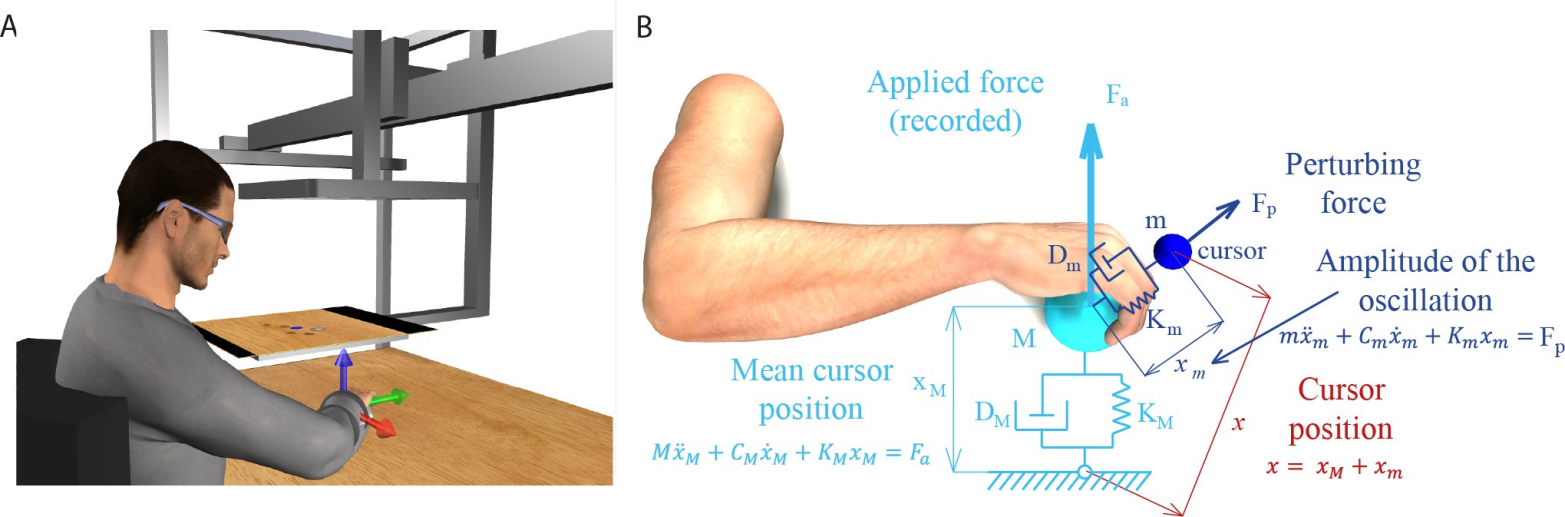
Experimental apparatus and cursor motion modeling. (**A**) Subjects sat in front of a desktop and inserted their right forearm into a splint connected to a 6 axis force transducer. The view of the hand was occluded by a mirror that reflected a virtual scene projected by a LCD monitor. The monitor displayed stereoscopically a desktop matching the real desktop and a blue spherical cursor whose position, when the subject was relaxed, was approximately at the center of the palm. The cursor position in space was simulated using two mass-spring-damper system and depended on the force applied by the subject. Adapted from [32]. (**B**) The position of the cursor was computed as the position of one mass (**x**_**m**_, mass m) connected through a spring (stiffness K_m_) and a damper (damping coefficient D_m_) to the position of a second mass (**x**_**M**_, mass M) connected to the center of the palm through another spring (stiffness K_M_) and damper (damping coefficient D_M_). The force applied isometrically by the hand to the splint (**F**_**a**_) was used to compute **x**_**M**_. Mass m oscillated around mass M while the position of mass M was not influenced by the position of mass m. The instantaneous muscle activation, projected onto the null space of the EMG-to-force matrix, was used to calculate the stiffness K_m_ and determined the amplitude of the oscillation of the cursor around its mean position (**x**_**M**_).

Subjects sat in front of a desktop on a racing car chair. Trunk and shoulders were immobilized by four-point safety belts and the right forearm was inserted into a splint that fully supported the hand and the forearm and immobilized the wrist joint. A steel rod connected the splint with a 6-axis force transducer (Delta F/T Sensor, ATI Industrial Automation, Apex, NC, USA) fixed below the desktop (Fig 1A). The position in the medio-lateral direction of the center of the palm in the splint was aligned with the body midline. The height of the desktop was adjusted such that the palm was at the height of the sternum. The distance between the desktop and the chair was adjusted to achieve an elbow flexion angle of 90°. This position was comfortable for the subjects and allowed them to remain still during the entire duration of the experiment. A mirror (29.7 x 21 cm), positioned parallel to the desktop approximately half-way between the hand and the eyes, occluded the hand. It reflected the image of a virtual scene displayed by a 21-inch LCD monitor (Syncmaster 2233, Samsung Electronics Italia S.p.A., Cernusco sul Naviglio, MI, Italy), parallel to the desktop and positioned approximately at the height of the eyes. During the experiment subjects wore 3D shutter glasses (3D Vision P854, NVIDIA Corporation, Santa Clara, CA, USA) and viewed stereoscopically a virtual scene reproducing the real desktop and a spherical blue cursor. The cursor appeared, at rest, approximately at the position of the center of the occluded palm. The virtual scene was rendered by a 3D graphic card (Quadro Fx 3800, NVIDIA) on a PC workstation, using custom software. The scene was updated at 60 Hz with the cursor position processed by a second dedicated data-acquisition PC workstation running a real-time operating system. The cursor position was transmitted to the first workstation through an Ethernet link using the UDP protocol.

Surface EMG activity was recorded from 17 muscles acting on the elbow and the shoulder: teres major (TeresMaj), infraspinatus (InfraSp), latissimus dorsi (LatDorsi), inferior trapezius (TrapInf), middle trapezius (TrapMid), superior trapezius (TrapSup), brachioradialis (BracRad), biceps brachii, long head (BicLong), biceps brachii, short head (BicShort), triceps brachii, lateral head (TriLat), triceps brachii, long head (TriLong), triceps brachii, medial head (TriMed), anterior deltoid (DeltA), middle deltoid (DeltM), posterior deltoid (DeltP), pectoralis major clavicular portion (PectClav), pectoralis major sternal portion (PectStern). The correct electrodes placement was verified by observing the activation of each muscle during specific maneuvers. EMG activity was recorded with active bipolar electrodes (DE 2.1, Delsys Inc., Boston, MA), after band pass filtering (20–450 Hz) and amplification (gain 1000, Bagnoli-16, Delsys Inc.). Both force and EMG data were digitalized at 1 kHz using an analog-to-digital PCI board (PCI-6229; National Instruments, Austin, TX, USA). Only force components were used to compute cursor motion (torque components were recorded but not used) and they were defined as: F_x_ as the component along the medio-lateral axis, positive to the right; F_y_ along the antero-posterior axis, positive away from the chest; F_z_ along the vertical axis, positive up.

The cursor position was computed in real-time using two mass-spring-damper systems (Fig 1B). At rest and without any perturbing force, the cursor was displayed at a position corresponding to the center of the palm. An isometric force (***F***_***a***_) applied by the subject to the splint displaced a first mass (*M*) connected through a spring and a damper to the position of the center of the palm (origin). The spring constant (*K*_*M*_) was set such that a constant force with a magnitude corresponding to 20% of the mean maximum voluntary force (MVF) across force directions (see below), would have maintained the cursor at 5 cm from the origin. The mass (*M*) was adjusted adaptively in the range 15–140 g as in [34] to reduce the end-point force fluctuation due to the muscle signal dependent noise. The damping constant (*D*_*M*_) was set to make the system critically damped. This mass-spring-damper system behaved like a low-pass filter for the mean motion of the cursor because it reduced the physiological high frequency fluctuations of the exerted forces, increasing during the co-contraction, and made the control of the cursor easier. The position of the cursor (***x***) corresponded to the position (***x***_***m***_) of a second mass (*m*) relative to the position of the first mass (***x***_***M***_). The two masses were connected by a spring and a damper whose stiffness (*K*_*m*_) and damping constant (*D*_*m*_) were adjusted in real-time according to level of muscle co-contraction expressed by the norm of the instantaneous projection of the muscle activation vector onto the null space of the EMG-to-force matrix (see below and Appendix).

### EMG-to-force matrix and its null space

The relation between the vector of recorded EMG signals (***m****)* and the recorded isometric end-point force ***F***_***a***_ was approximated as linear, $F_{a}=H∙m$, where the EMG-to-force matrix (***H***) was estimated in using multiple linear regression. The dimensions of ***H*** were $n_{d}\times n_{m}\;(n_{d}=3$ number of space dimensions, $n_{m}=17$ number of recorded muscles). The EMG signals from all muscles, after rectification, baseline subtraction, filtering, re-sampling at 100 Hz, normalization by the maximum voluntary contraction (MVC) level, were regressed on each force component (2^nd^ order Butterworth low-pass filtered, 5 Hz cutoff) recorded during the hold phase in all baseline trials. Despite the relationship between muscle activation and end-point isometric force is generally non-linear, a linear relationship provides an adequate approximation for low muscle activation levels, as the one required to reach the 20% MVF targets in our experiment. The quality of the reconstruction of the forces, recorded during the static phase in the baseline condition (see below) was used to assess the validity of the linear approximation in each participant. The R^2^ value of the reconstruction was higher than 0.76 in all participants except one, who presented a R^2^ equal to 0.61, and was thus excluded from the analysis. The mean R^2^ value of the remaining participants (labeled as Subject 1 to 8) was 0.83 ± 0.05 (std, n = 8).

The set of muscle activation vectors that did not generate any end-point force, forming a subspace of the muscle activation vector space, was estimated computing the null space matrix ***N*** of the EMG-to-force matrix ***H*** with the Matlab function *null*. The dimensions of ***N*** were $n_{m}\times (n_{m}-n_{d})$. The instantaneous projection of the muscle activation vector in the null space vector ***n***, used to set the stiffness *K*_*m*_ and the damping *D*_*m*_ (see Appendix), was calculated at each time sample *i* as the product of the transposed null space matrix by the EMG activation vector: $n_{i}=N^{T}m_{i}$.

### Experimental protocol

**Fig 2:**
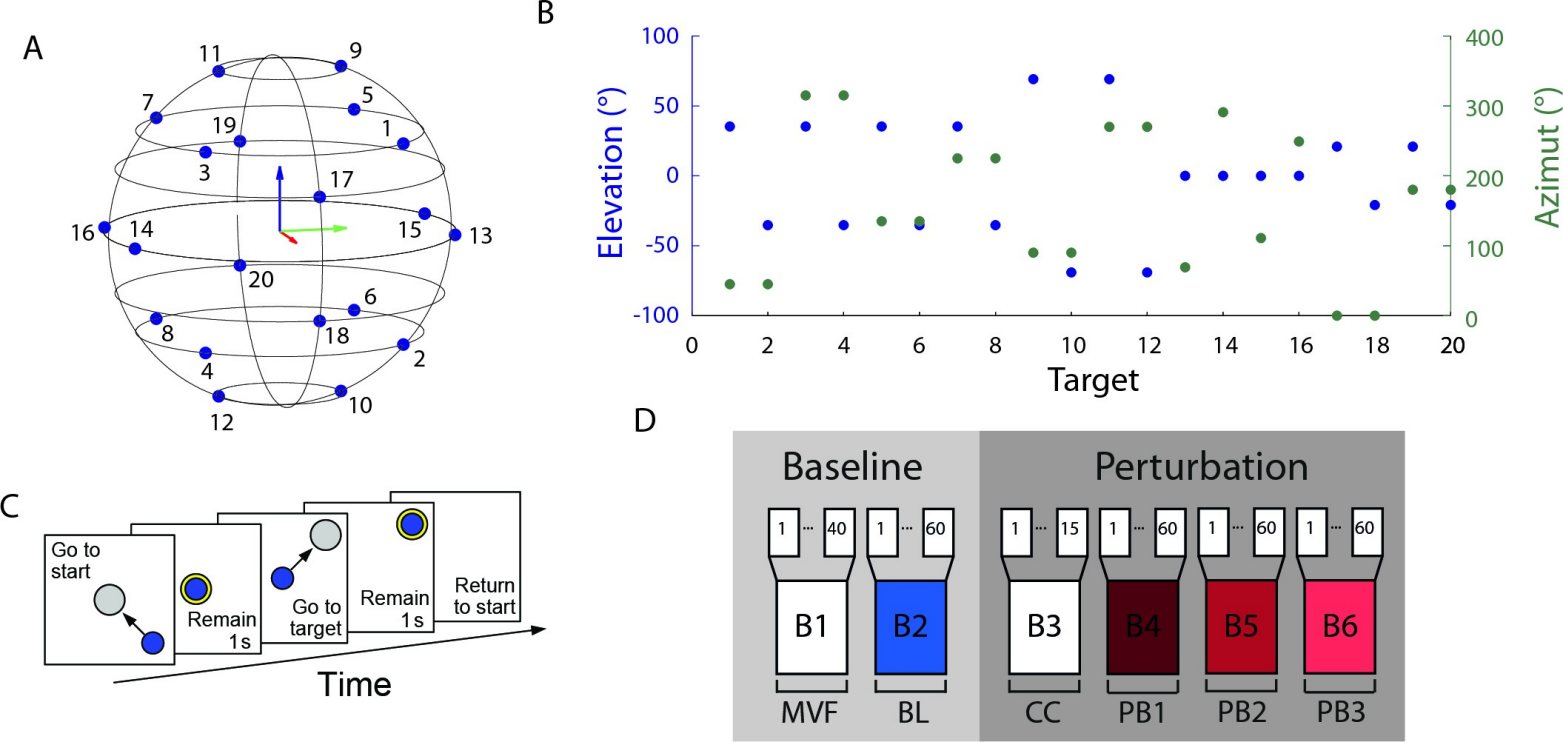
Experimental protocol. (**A, B**) Targets were equally distributed on the vertices of a dodecahedron inscribed in a sphere whose radius was 20% MVF. (**C**) Subjects were instructed to perform a reaching task. At the beginning of the trial subjects had to maintain the cursor in the central location for 1 s. When a transparent gray sphere appeared in one of the target positions, subjects had to apply a force to move the cursor inside the sphere. The color of the sphere switched from gray to yellow when the cursor was fully inside the larger target sphere. Subjects had to match the force target with a tolerance of 3% MVC to maintain the cursor inside the sphere for 1 s (hold phase). Finally, subjects had to relax all muscles to make the cursor return to the initial position. (**D**) Each subject performed a single experimental session consisting of 40 trials of maximum voluntary force generation along the 20 directions (B1), 60 trials of reaching to targets along the 20 directions (B2), 15 trials of pure co-contraction (B3), and 3 blocks of 60 reaching to targets trials along the 20 directions with three different levels of perturbation magnitude (B4, B5, B6).

The experiment was subdivided in 6 blocks (Fig 2D), each consisting in a sequence of trials. In the first block (B1, MVF block) subjects had to generate maximum voluntary force in 20 different target directions twice (40 trials). The target directions were defined as the directions of the vertices of a dodecahedron with respect to its center in the origin (Fig 2A). In each trial the subjects saw an arrow starting from the origin and with a length and direction corresponding to the magnitude and direction of the applied force. The target force direction was displayed as a transparent gray cylinder. Subjects were instructed to apply a maximal force in the target direction and to maintain it for 1 s. A new trial started in a different direction 8 s after the end of the previous trial. Data collected from this block were used to establish the mean MVF across all directions of the maximum force applied. Forces were filtered by a second order Butterworth filter with a 5 Hz low-pass cutoff. During the second block (B2, baseline block), subjects had to displace the cursor to reach one of 20 targets. Targets were positioned at the vertices of a dodecahedron inscribed in a sphere centered at the origin and whose radius was 20% MVF (Fig 2A and 2B). Each target was presented 3 times, for a total of 60 trials. At the beginning of each trial, subjects were instructed to remain relaxed (rest phase) and maintain for 1 s the cursor inside a sphere centered at the origin and whose radius was 2% MVF larger than the radius of the cursor (Fig 2C). During the rest phase EMG signals collected from each muscle (rectified, filtered, re-sampled, baseline subtracted, MVC normalized) were averaged and their means, identified as baseline noise, were subtracted from the EMG signals collected during the rest of the trial. At the end of the rest phase a transparent gray sphere, whose radius was 3% MVF larger than the radius of the cursor, was displayed in one of the 20 possible target locations, randomly selected in each repetition. Subjects were instructed to apply a force to displace the cursor from the origin to the target, and to maintain it within the target sphere for 1 s (hold phase) for successfully completing the trial. The target color switched from gray to yellow when the cursor was inside it. Subjects were required to complete the trial within 15 s. The baseline block was used to calculate both the EMG-to-force matrix and the mean norm of the muscle activation projected onto the null space, which would be used to normalize the norm of the null space projection in the following blocks (see Appendix).

The third block (B3, pure co-contraction block) was introduced to familiarize subjects with the co-contraction task; it was composed of 15 trials in which the target sphere was positioned at the origin. At the end of the baseline block, subjects were informed that an oscillation would perturb the cursor in the following trials and that they could reduce it by stiffening their arm. The action of the perturbation on the cursor started after the end of the rest phase and finished with the end of the hold phase. Subjects were required to maintain the cursor, which was perturbed with a noise force of magnitude level 2 (see Appendix), within the target (3% MVF tolerance) during the 1s hold phase. A time-out of 15 s was used to avoid fatigue if subjects were not able to maintain the cursor in the target for the requested time. In the last three blocks (B4, B5, and B6, perturbed blocks) subjects had to reach one of 20 targets positioned at the vertices of a dodecahedron inscribed in a sphere of 20% MVF radius and centered at the origin. Each target was presented three times for a total of 60 trials in each block. The cursor was perturbed by a disturbing force (see Appendix) with a magnitude of level 1 in B4, level 2 in B5, and level 3 in B6. A trial was successful if the cursor remained inside the target for 1 s and a 15 s time-out was used to avoid fatigue. Two breaks were scheduled. The first break was necessary, after the baseline block, to allow for the automatic processing of the data for the calculation of the ***H*** and ***N*** matrixes, and to explain the co-contraction task to the subject. The second break was scheduled after the fifth block to allow subjects to rest. In addition to the scheduled breaks, subjects could pause at any time.

### Data analysis

EMG directional tuning curves, their fits with a spatial cosine function, and the projection of the muscle activation in the null space were calculated and compared among blocks. Since the muscle activations for all muscles in each trial can be described as a vector in muscle space, the angle between muscle activation vectors recorded during different blocks were calculated. A few trials (19.1 ± 12.6, mean ± SD across all subjects, over 195 trials performed by each subject) were excluded from the analysis after visual inspection because of: 1) high level of noise contaminating the EMG signal likely due to suboptimal contact of the electrodes with the skin; 2) artefacts in the EMG signal likely due to accidental contact of the subjects with metallic elements of the apparatus; 3) transients in the force likely due to accidental impact of the subject’s left arm with the force transducer; 4) muscle activity during the rest phase. A trial-specific baseline noise level was estimated as the mean activation of each muscle recorded at the beginning of each trial (rest phase). Data recorded during the last 0.4 s of rest phase of each trial, in which movements anticipation may occur, were excluded in the evaluation of the trial-specific noise level. The trial-specific baseline noise was subtracted from the rest of the data of each trial.

#### Task performance

A trial was successful if the cursor remained inside the target sphere (3% MVF tolerance) during the 1 s hold phase. In successful trials, we defined *time-to-criterion* as the interval from target appearance to the beginning of the hold phase. In unsuccessful trials, for which no hold phase could be defined, the time-to-criterion was defined as the interval from target appearance to the last time in the trial in which the cursor entered the target. The time interval between the first moment the subject reached the target and the end of the hold phase, in successful trials, or the end of the trial in unsuccessful trials, was defined as *attempting time*. The attempting time represents the time the subject tried, successfully or not, to maintain the cursor inside the target. The norm of the difference between the applied force, normalized to MVF, averaged during the attempting time, and the force target was defined *force error*. The norm of the difference between the cursor position displayed to the subject, and the position of the target was calculated and converted to force units through the stiffness of the first mass-spring-damper system. This difference, normalized to MVF, averaged during the attempting time, was defined as *cursor error*.

#### Directional tuning of muscle activations

EMG waveforms were rectified, digitally low-pass filtered (zero-phase, second order Butterworth, 5Hz cutoff), and re-sampled at 100 Hz to reduce data size. The rectified and filtered EMG signals for each muscle were normalized to the maximum voluntary contraction across directions (MVC) recorded during the MVF block. The waveforms of each muscle were averaged during the hold phase and the mean values recorded during the trials of the same block and with the same target direction, were averaged to construct the directional tuning curves.

The directional tuning of each muscle, calculated separately from each block, was fitted by a spatial cosine function, defined by four parameters: azimuth $\vartheta_{PD}$, elevation $\varphi_{PD}$, amplitude $f_{PD}$, and offset $m_{offset}$. These parameters were estimated with a multiple linear regression (Matlab function *regress*). The equation that defined the spatial cosine function for each muscle was [32]:

$$m\left(f,f_{PD},m_{offset}\right)=f^{T}\cdot f_{PD}+m_{offset}=f_{PD}\left(\text{cos}\varphi \cdot \text{cos}\varphi_{PD}+\text{sin}\varphi \cdot \text{sin}\varphi_{PD}\cdot \text{cos}\left(\vartheta -\vartheta_{PD}\right)\right)+m_{offset}$$

where ***f*** is the unit vector pointing in the force direction, defined by azimuth and elevation angles ϑ and $\varphi ,f_{PD}$ is the preferred direction vector. We considered a muscle to be cosine tuned if the regression was significant (*p < 0*.*05*) and the quality of the fit acceptable (*R*^*2*^ *> 0*.*5*). Only muscles whose directional tuning was cosine tuned in the baseline and in at least one perturbed block were compared.

#### Null space components

**Fig 3:**
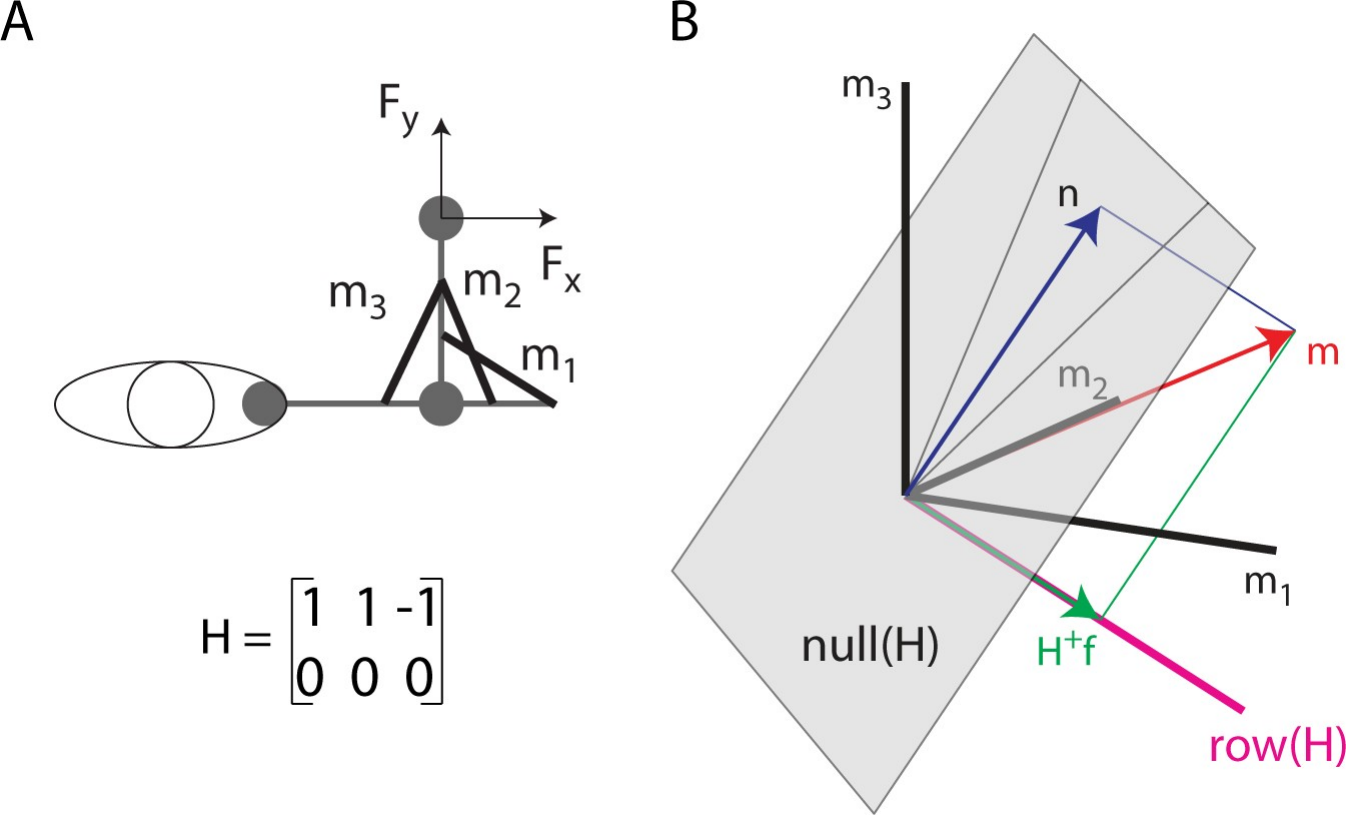
Illustration of the decomposition of a muscle vector onto the null space and the row space of the EMG-to-force matrix. (A) Toy model of a 2 DOF arm with three muscles (m_1_, m_2_: elbow extensors; m_3_: elbow flexor) generating force along the x-axis according to the specified EMG-to-force matrix (***H***). (B) A muscle pattern ***m*** (in the [2 2 1]^T^ direction) is illustrated as a vector (*red arrow*) in a three-dimensional space (coordinate axes: *black lines*) together with its projection ***n*** (*blue arrow*) onto the *null space* of the ***H*** matrix (*gray plane*) and its projection ***H***^***+***^***f*** (green arrow) onto the *row* (or *force*) *space* of the ***H*** matrix (*magenta line*, in the [1 1 –1]^T^ direction). Because the force space has a negative m_3_ component, the minimum norm solution of the ***F*** = ***H m*** equation (***H***^***+***^***f***) cannot be achieved with non-negative muscle activations and the non-negative muscle vector ***m*** is generated adding a null space vector component (***n***).

The *muscle space* is defined as the space whose coordinates are the activations of each muscle, so its dimensionality is the number of EMG signals recorded. In this space, the activation of all muscles recorded at a specific time sample is represented by a vector. Each vector (i.e. each muscle activation pattern) can be decomposed into two vectors obtained by projection onto two orthogonal subspaces [35,36]: the *null space* of ***H***, whose elements are mapped by the ***H*** matrix onto zero end-point force, and the row space of ***H*** or *force space*, whose elements are mapped by the ***H*** matrix onto non-zero end-point force. Any muscle activation vector can be thus uniquely decomposed into two orthogonal vectors by projection onto these two subspaces (Fig 3). The null space projection was computed with the Matlab function *null* and the force space projection was computed as the Moore-Penrose pseudo-inverse of the ***H*** matrix (***H***^***+***^) with the Matlab function *pinv*. However, the pseudo-inverse does not necessarily provide a physiological muscle vector ***m*** that generates a desired force ***F***. In fact, ***m***_***min***_ = ***H***^***+***^ ***f*** is the solution to the underdetermined system of equations ***f*** = ***H m*** with minimum norm but ***m***_***min***_ may have negative components. To solve the ***f*** = ***H m*** equation with the physiological constraint of non-negative muscle activations, i.e. *m*_*i*_ ≥ 0 for all *i* = 1…*n*_*m*_, in general it is necessary to add to ***m***_***min***_ an appropriate null space vector ***n***.

**Fig 4:**
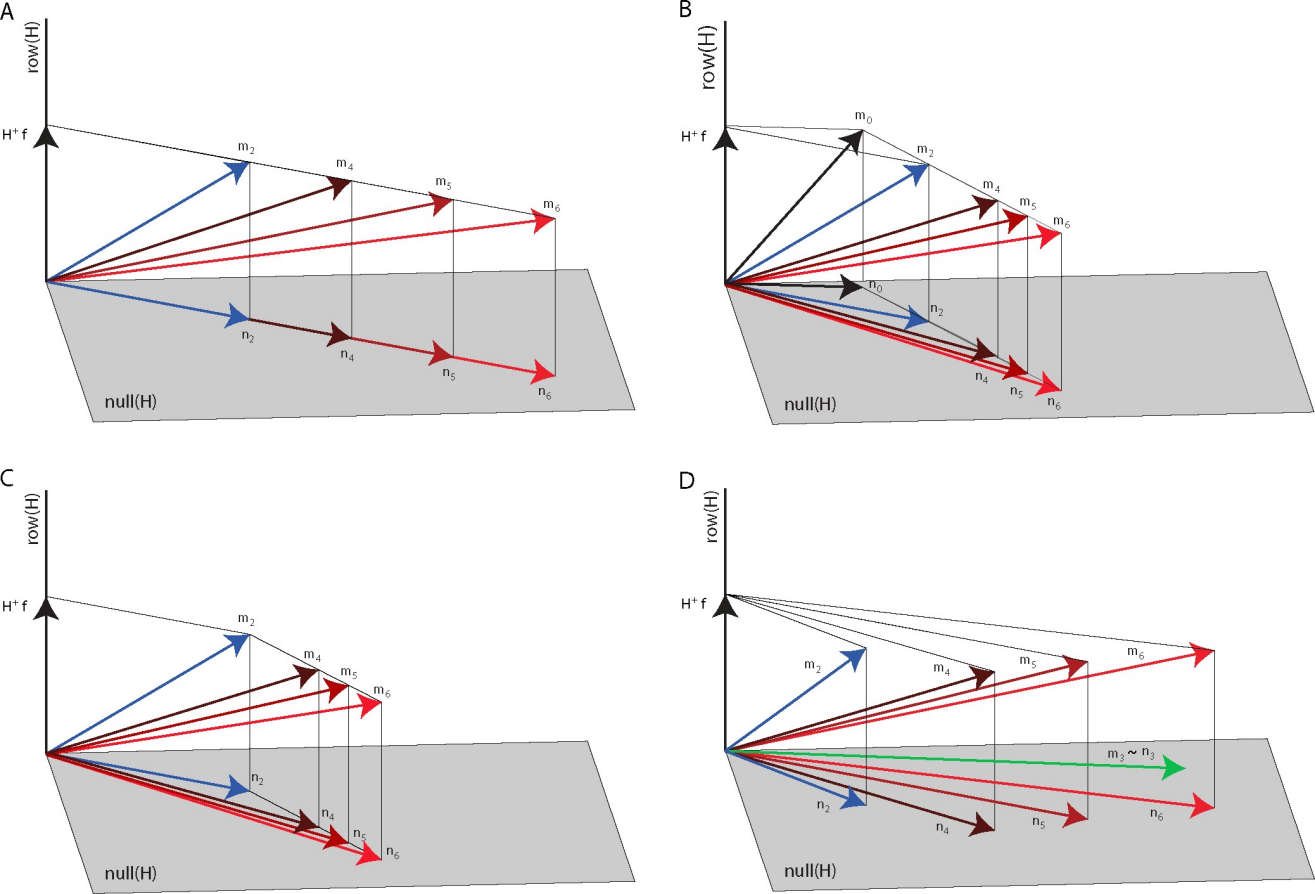
Null space modulation with co-contraction. Four hypotheses on the changes of the null space vector with an increase of co-contraction are illustrated in a three-dimensional muscle space with a two-dimensional null space (*gray plane*) and a one-dimensional row or force space (*black line*) orthogonal to the null space. (A) According to the first hypothesis, the null space vector (***n***_***2***_, *blue arrow*) associated with the baseline block muscle vector (***m***_***2***_, *blue arrow*) is scaled in amplitude to increase co-contraction (***n***_***4***_, ***n***_***5***_, ***n***_***6***_, *red arrows*) to generate the muscle vectors in the perturbed blocks (***m***_***4***_, ***m***_***5***_, ***m***_***6***_, *red arrows*, all generating the same force vector ***f***). Thus, ***n***_***4***_, ***n***_***5***_, and ***n***_***6***_ are collinear with ***n***_***2***_. (B) According to the second hypothesis, the null space vector (***n***_***2***_, *blue arrow*) associated with the baseline block muscle vector (***m***_***2***_, *blue arrow*) is generated adding a null space vector (***n***_***2***_ –***n***_***0***_) to the minimum-norm non-negative muscle vector (***m***_***0***_) generating the target force (***f***) and such vector is scaled in amplitude to increase co-contraction. Thus, the difference between the null space vectors in the perturbed blocks (***n***_***4***_, ***n***_***5***_, ***n***_***6***_) and ***n***_***0***_ are collinear with (***n***_***2***_ –***n***_***0***_). (C) According to the third hypothesis, the null space vector of the perturbed blocks (***n***_***4***_, ***n***_***5***_, ***n***_***6***_, *red arrows*) associated with the perturbed blocks muscle vector (***m***_***4***_, ***m***_***5***_, ***m***_***6***_, *red arrows*) is generated adding a null space vector (***n***_***i***_−***n***_***2***_ with ***i = 4*, *5*, *6***) to the muscle vector recorded during the baseline (***m***_***2***_) generating the target force (***f***). Such vector is scaled in amplitude to increase co-contraction. Thus, the difference between the null space vectors in the perturbed blocks (***n***_***4***_, ***n***_***5***_, ***n***_***6***_) and ***n***_***2***_ are collinear with each other but are not collinear with ***n***_***2***_ or (***n***_***2***_ –***n***_***0***_). (D) According to the fourth hypothesis, the null space vectors of the perturbed blocks (***n***_***4***_, ***n***_***5***_, ***n***_***6***_, *red arrows*) associated with the perturbed blocks muscle vector (***m***_***4***_, ***m***_***5***_, ***m***_***6***_, *red arrows*) are generated as a linear combination of the null space vector of the baseline block (***n***_***2***_, blue *arrow*) associated with the baseline block muscle vector (***m***_***2***_, *blue arrow*) and the pure co-contraction block muscle vector (***m***_***2***_, *green arrow*).

Since muscle activations generating the same end-point force have the same projection in the force space, two muscle activation vectors with different levels of co-contraction only differ for their projection in the null space. Thus, the CNS modulates co-contraction when generating a given endpoint force by varying the projection of the muscle activation in the null space. For a given force target, different levels of co-contractions can be achieved by many different choices of the null space component. A vector scaling strategy consists in changing the amplitude of the null space component without changing its direction (hypothesis 1, Fig 4A). Indeed, with a scaling strategy the level of co-contraction, and thus the magnitude of end-point impedance, may be controlled by a single parameter, a scaling coefficient. Such scaling strategy can be detected by checking the cosine of the angle between the null space projections of muscle activations with different levels of co-contraction. This cosine should remain close to 1, despite the physiological EMG variability whose effect we estimated (see Statistical analysis below) by computing the angles between the null space projections of muscle activations recorded during different repetitions of the same baseline target.

The baseline null space projection of the muscle activation vector may not be generated according to an explicit impedance control strategy but rather be a consequence of the fact that physiological muscle activations are non-negative. Moreover, subject might minimize effort while achieving the primary goal of generating a desired force. Thus, we also computed, for each force target, the minimum-norm non-negative muscle activation $m_{0}$:

$$m_{0}=\arg\;min(||m||)\;\text{such}\;\text{that}\;f=H\cdot m\;\mathrm{and}\;m_{i}\geq 0\;\mathrm{with}\;i=1\ldots n_{m}$$

where ***f*** is the applied force and $n_{m}$ the number of muscles. The Matlab function *quadprog* was used to calculate these minima. We then tested an alternative affine scaling strategy considering that the CNS might generate co-contraction patterns starting from the minimum-norm non-negative muscle vector ***m***_***0***_ and increasing null space activation along a unique direction (hypothesis 2, Fig 4B). We thus considered the differences between the muscle activation vectors (**m**) observed for each force target in different blocks and $m_{0}$ and we tested whether increased co-contraction levels were achieved by scaling ${m-m}_{0}$ which is, by construction, a null space vector. Angles between ${m-m}_{0}$, recorded during trials with the same endpoint force target and different co-contraction levels were calculated. The cosine of the angles between ${m-m}_{0}$ calculated during different blocks should be close to 1, despite the physiological EMG variability whose effect we assessed, as for the first hypothesis, by computing the angle between the ${m-m}_{0}$ vectors calculated during different repetitions of the same baseline target.

We also tested whether the CNS used an affine scaling law obtained by scaling the difference of the muscle vector observed in each perturbed condition and the mean muscle vector observed, for each target, in the baseline condition ($m-{\bar{m}}_{2}$) (hypothesis 3, Fig 4C). In this case the cosine of the angles between $m-{\bar{m}}_{2}$calculated during different perturbed blocks should be close to 1, despite the physiological EMG variability whose effect we estimated by computing the angle between $m_{2}-{\bar{m}}_{2}$ in each baseline repetition respect with the mean among the baseline repetitions (${\bar{m}}_{2}$).

Finally, we tested an additional hypothesis involving the mean muscle activation vector recorded during the pure co-contraction block (${\bar{m}}_{3}$ or equivalently ${\bar{n}}_{3}$ as such vector does not generate any actual force and thus belongs to the null space of **H**). For each target, the null space projection of the muscle vector observed in each perturbed condition (***n***_***4***_, ***n***_***5***_, ***n***_***6***_) might be obtained by a linear combination of the null space projection of the muscle vector in the baseline condition (***n***_***2***_) and the pure co-contraction vector ${\bar{m}}_{3}$ (hypothesis 4, Fig 4D). In this case the cosine of the angles between the null space vectors ${\bar{n}}_{i}$ (averaged over repetitions during different perturbed blocks, i = 4,5,6) and the subspace spanned by ${\bar{n}}_{2}$ and ${\bar{m}}_{3}$ should be close to 1, despite the physiological EMG variability whose effect we estimated by computing the angle between the null space projection of different repetitions of the baseline vectors ($n_{2})$ with respect to the subspace spanned by the mean of the projection of the other repetitions and ${\bar{m}}_{3}$. The EMG data used to test the four hypotheses were obtained by averaging the time samples in the static phase.

#### Statistical analysis

Repeated measures ANOVA was performed on time-to-criterion, force and cursor errors, amplitude and offset of the muscle activation cosine tuning with block as factor (4 levels: baseline B2 and three noise levels B4, B5, B6) to assess the effect of the perturbation. Post-hoc tests were performed to check the relationships between different blocks, based on the ANOVA outcomes. To this end multiple comparisons were carried out via six t-tests comparing the distributions in each pair of experimental blocks. Due to the unequal sample size between blocks the Welch Two Sample t-test was applied.

The effect of the perturbation on the preferred directions angles of cosine tuning was tested comparing the cosines of the angles between preferred directions of all pairs of 4 blocks (6 levels). As Kolmogorov-Smirnov tests indicated that the cosines of the angles were not normally distributed, the Kruskal-Wallis (KW) non-parametric test was used to compare the medians across block pairs. Post-hoc Bonferroni corrections were applied to post-hoc comparisons.

Assuming that the muscle patterns in different repetitions of the same baseline target were generated according to the same co-contraction strategy, i.e. with collinear null space vectors, we used the observed directional variability of the muscle activation vectors recorded during different repetitions of the same baseline target to estimate the effect of the physiological variability and EMG noise on the assessment of the collinearity between null space vectors among different blocks. Thus, we used the distribution of the values of the cosine of the angles between null space vectors recorded during different repetitions in the baseline block to assess whether a cosine value smaller than 1 between perturbed and baseline nulls space vectors was significantly different from the value expected in presence of only physiological variability and EMG noise, indicating a deviation from collinearity.

To test the first hypothesis on null space vectors scaling, we computed the angles between the null space projections of each target repetition of perturbed blocks trials with respect to each repetition of baseline block trials for the same target, and we compared its distribution with the distribution of the angles among each target repetition of the null space projections of baseline trials.

To test the second hypothesis on null space vectors scaling, we computed the difference between the observed muscle activation vector (**m**_**i**_, i = 2 for baseline, i = 4,5,6 for perturbed blocks) and the non-negative minimum-norm muscle activation vector (**m**_**0**_) for each target. The distribution of the angle between all the repetitions of the **m**_**4,5,6**_-**m**_**0**_ calculated during the perturbed blocks and all the repetitions of the **m**_**2**_-**m**_**0**_ calculated during the baseline block was compared with the distribution of the angle among the different repetitions of the **m**_**2**_-**m**_**0**_ calculated during the baseline block.

To test the third hypothesis on null space vectors scaling, we subtracted the muscle activation vector, recorded during the baseline block and averaged among the repetitions of the same target, from each repetition of the muscle activation recorded during the perturbed blocks ($m_{p}-{\bar{m}}_{2}$). Its distribution was compared with the distribution of the angle among the different repetitions of the $m_{2}-{\bar{m}}_{2}$ calculated during the baseline block.

Finally, to test the fourth hypothesis, for which the generation of null space vectors when additional co-contraction was required was achieved as linear combination of the null space component of the muscle vector recorded during the baseline condition (force-only) and the muscle vector recorded during the pure co-contraction condition, for each target, we computed the angle between the mean null space projection (averaged over repetitions) of the muscle activation vector of each perturbed block (${\bar{n}}_{i}$, i = 4,5,6) and the subspace spanned by the mean null space projection of force-only muscle activation vector (${\bar{n}}_{2}$) and mean muscle activation vector recorded during the pure co-contraction block (${\bar{m}}_{3}$). To assess the deviation for a perfect alignment with the subspace due to physiological variability and EMG noise, for each target, we computed the angle between the null space projection of the muscle vectors for each repetition in force-only condition and the subspace spanned by the mean of the null space projection of the remaining repetitions and the mean muscle activation vector in the pure co-contraction condition, and we averaged the angles obtained for the different repetitions. Then, for each perturbed block, the distribution of the angles for all targets was compared with the distribution of the angles due to noise.

For all four tests, a Wilcoxon singed rank test was used to test the statistical difference, with a p-value threshold of 0.05.

## Results

**Fig 5:**
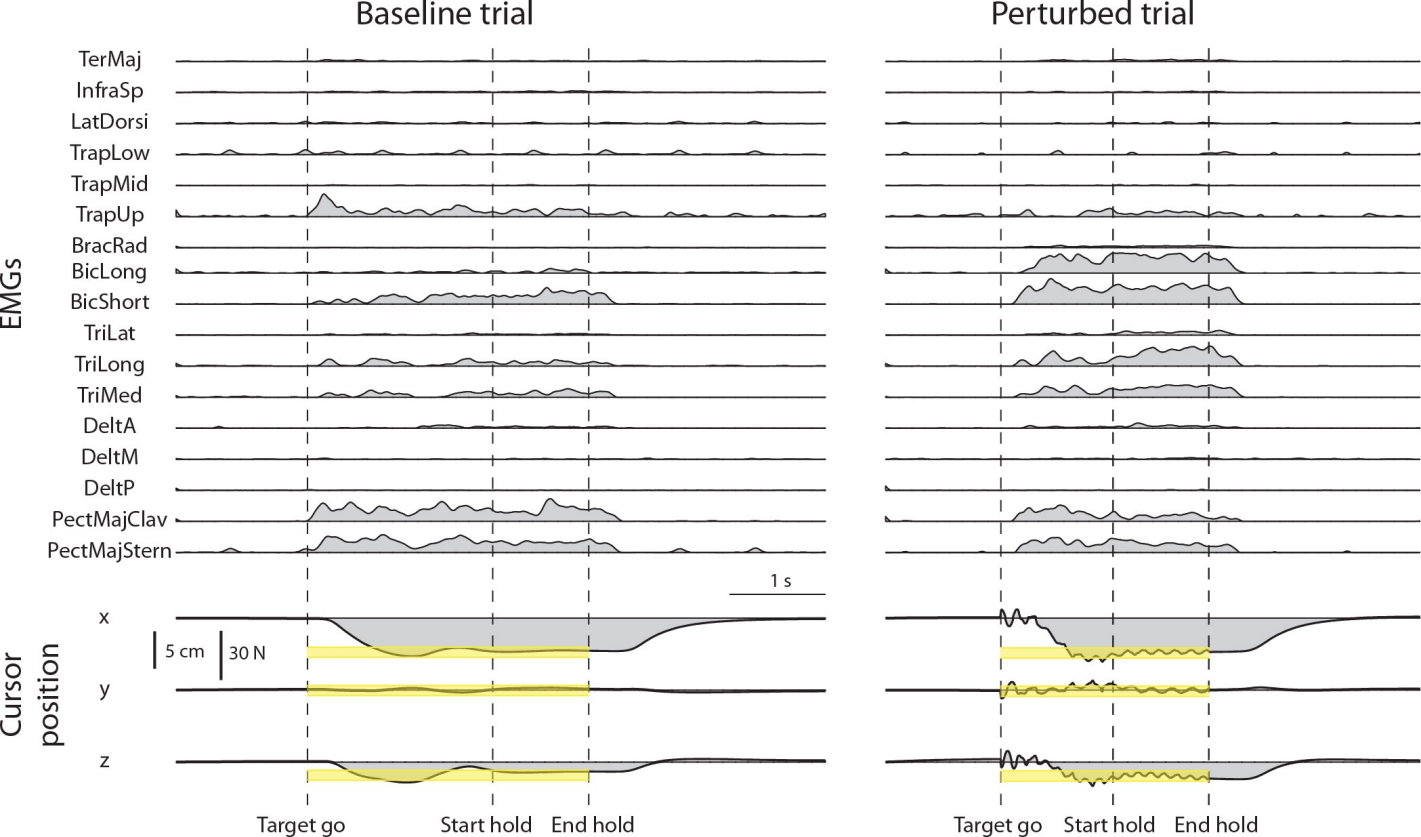
Examples of muscle activity and cursor position time-course in a baseline and a perturbed trial. A trial of the baseline block (left) and a trial of the perturbed block with perturbation magnitude level 2 (right) for the same force target (target 20 of Fig 2) are shown. EMG data were rectified, low-pass filtered, resampled, baseline subtracted, and normalized to the MVC of each muscle. Cursor position was resampled. Targets were positioned at 20% MVF (corresponding to a displacement of 5 cm from the origin) with a tolerance around the target of 3% MVF (yellow bands). The dashed vertical lines indicate the time of target appearance (Target go), the last time of the trial in which the cursor entered the target (Start hold), and the end of the hold phase, i.e. the 1s interval in which the cursor remained in the target (End hold). In perturbed blocks (right) the oscillation of the cursor started at Target go and ended at End hold.

All subjects could reach and maintain the cursor within each target with the required 3% MVF tolerance when no perturbation was applied to the cursor (baseline). All subjects could also reach each target and increase co-contraction to maintain the cursor within the target with the required 3% MVF tolerance during at least one trial of each perturbed block. Examples of rectified, filtered, resampled, baseline subtracted, and MVC normalized EMG signals are shown in Fig 5 (*top*) together with the cursor position (*bottom*) for a baseline trial (*left*) and a perturbed trial (noise level 2, *right*).

### Task performance

**Fig 6:**
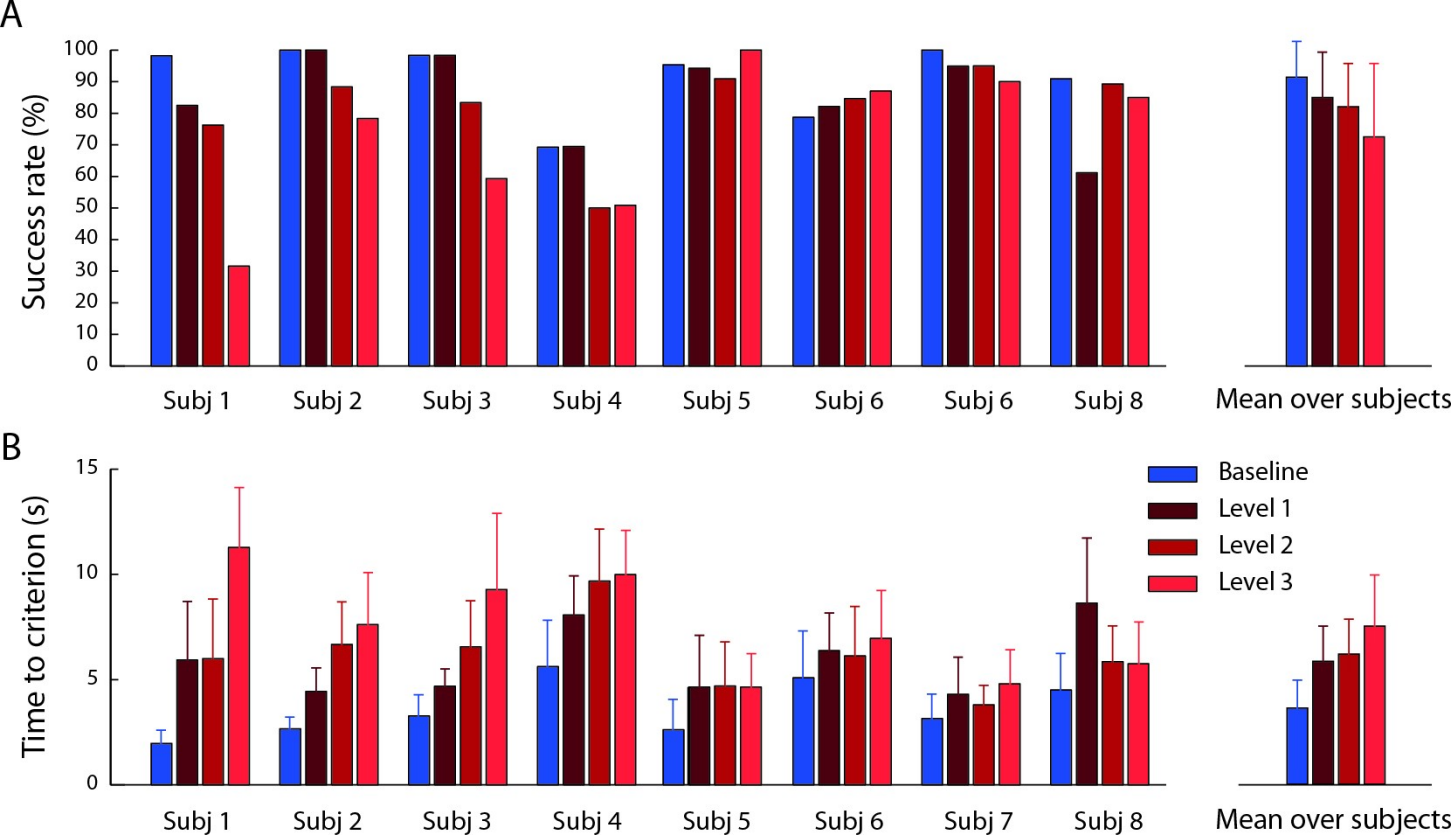
Task performance. (A) Success rate. The mean fraction of successful trials is shown for each subject and each noise condition together with its mean among subjects. The bars represented the percentage of selected trials that were successful (subjects were able to remain within the target for 1 s). The color indicated the noise level (blue: no noise, dark red: noise level 1, medium red: noise level 2, light red: noise level 3). (B) Time-to-criterion (mean ± SD) for each subject and noise condition together with its mean among subjects. Time-to-criterion was the time required by the subject to reach and hold the target position. It corresponded to the beginning of the hold phase for successful trials and to the last time the subject got inside the target for the non-successful trials.

The number of selected successful trials was: 46.9 ± 9.3 (mean ± SD across subjects, see individual performance in Fig 6A) over 60 baseline trials, 11.1 ± 3.9 over 15 pure co-contraction trials, 48.7 ± 9.1 over 60 perturbed trials of noise level 1, 46.4 ± 9.0 over 60 perturbed trials of noise level 2, 42.4 ± 13.5 over 60 perturbed trials of noise level 3. The time required to reach and hold the target position (time-to-criterion, Fig 6B) increased in the perturbed blocks. The ANOVA performed on time-to-criterion showed a significant effect of the block factor (F_1, 1770_ = 243.7, p < 0.001). Post-hoc comparisons (Welch t-test) indicated that significant differences were present between the baseline block and all perturbed blocks and between all pairs of perturbed blocks (B2-B4: t(812.8) = -10.8, p <0.001; B2-B5: t(773.8) = -11.5, p <0.001; B2-B6: t(742.6) = -16.3, p <0.001; B4-B5: t(895.8) = -1.11, p = 0.27; B4-B6: t(882.3) = -6.3, p <0.001; B5-B6: t(899.7) = 5.1, p <0.001).

**Fig 7:**
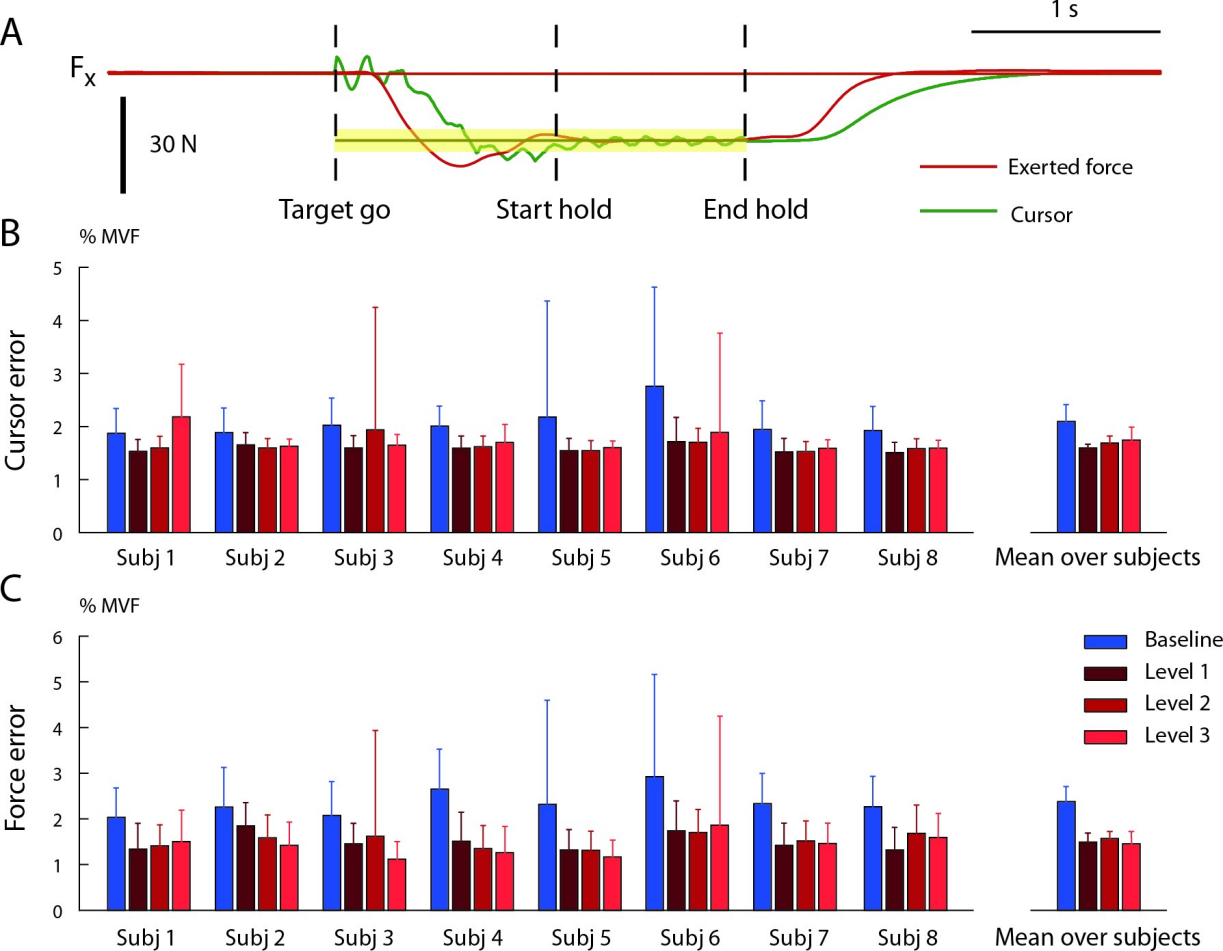
Cursor position and exerted force errors. (A) Example of the actual force exerted by the subject (red), used to calculate the force error, and the feedback of force given as the cursor displacement (green), used to calculate the cursor error, along the x component. The trial is the same as in the right side of the Fig 5. The target position is in dark yellow and the tolerance in yellow. (B) The cursor position was converted into the force exerted by the subject. This cursor force differed from the force applied by the subject because of the perturbation. The figure represents the norm of the difference between the force related to the cursor position and the target force, averaged during the hold time and normalized to MVF, for different blocks and subjects (mean ± SD across trials). On the right side the cursor error was averaged among subjects (mean ± SD across subjects). Blue bars are baseline block (no noise), red bars represent the perturbed blocks with different noise levels (noise 1 dark red, noise 2 medium red and noise 3 light red). (C) Norm of the difference between the force applied by the subject at the endpoint and the target force, averaged during the hold time and normalized to MVF, for different blocks and subjects (mean ± SD across trials). On the right side, the force error was averaged among subjects (mean ± SD across subjects). Blue bars are baseline block (no noise), red bars represent the perturbed blocks with different noise levels (noise 1 dark red, noise 2 medium red and noise 3 light red).

The comparison between force and cursor errors provided an indication of the perturbation compensation strategy used by each subject. Subjects could succeed in the task if they managed to combine a reduction of the cursor oscillation, achieved by increasing co-contraction, and an increase in the accuracy of the end-point force exertion. Thus, the required accuracy could be obtained with different combinations of co-contraction and force accuracy increments. The mean cursor position error (Fig 7B) reflects the combination of both force accuracy and co-contraction, while the mean force error (Fig 7C) only reflects force accuracy. The difference between the cursor error and the force during baseline block was due to the filtering effect of the first mass-spring-damper system. Significant effects of the block factor were observed both for the force error (F_1,1778_ = 209.7, p < 0.001) and the cursor error (F_1, 1778_ = 45.4, p < 0.001). Post-hoc comparisons (Welch t-test) indicated that significant differences in the force error were present between the baseline block and all perturbed blocks but not between perturbed blocks (B2-B4: t(549.7) = 12.4, p < 0.001; B2-B5: t(743.1) = 10.75, p < 0.001; B2-B6: t(754.9) = 12.01, p < 0.001), B4-B5: t(737.4) = -0.47, p = 0.6; B4-B6: t(753.6) = 1.48, p = 0.13; B5-B6: t(914) = -1.62, p = 0.11). Post-hoc comparisons (Welch t-test) indicated that significant differences in the cursor error were present between the baseline block and all perturbed blocks, between B4 and B6, but not between B4 and B5 and between B5 and B6 (B2-B4: t(456.6) = 8.8, p < 0.001; B2-B5: t(767.9) = 6.53, p < 0.001; B2-B6: t(730.9) = 5.3, p < 0.001; B4-B5: t(545.6) = -1.25, p = 0.212; B4-B6: t(584.5) = -3.7, p < 0.001; B5-B6: t(903.1) = -1.70, p = 0.09). Thus, subjects implemented a strategy that reduced the applied force error and performed the force-reaching task more accurately with the noise perturbation; i.e., both force and impedance adaptation occurred, similarly to what observed when learning to reach in a divergent force field [8].

### Muscle activation and its projection in the null space

**Fig 8:**
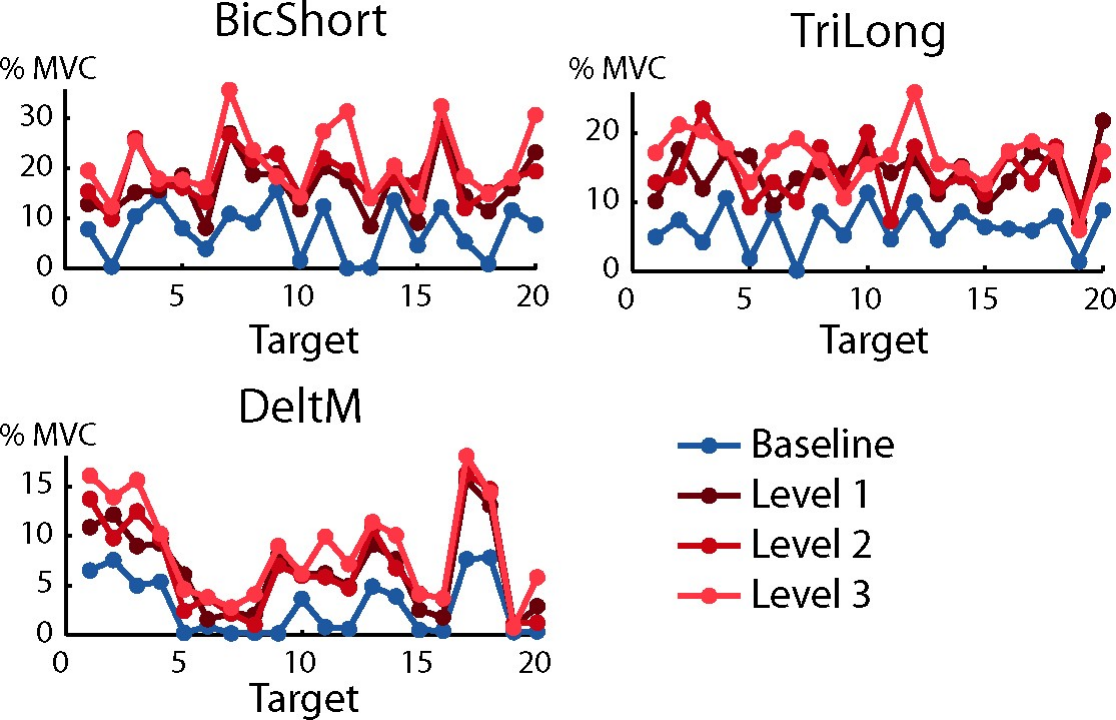
Examples of directional tuning of muscle activations. The mean muscle activation of three muscles recorded from subject 7 are shown as a function of target number (see Fig 2A and 2B) in baseline (*blue*) and perturbed blocks with perturbation magnitude level 1 (*dark red*), level 2 (*medium red*) and level 3 (*light red*). Note that the targets, defined in Fig 2A, are arranged at the vertices of a dodecahedron. Muscle activation is expressed as the fraction of the MVC recorded during the MVF block (B1).

As in previous studies [32,37,38,39] the activation of most muscles was modulated by force target direction. Fig 8 shows the dependence of the activation of three muscles, recorded in subject 8 during baseline (*blue*) and perturbed blocks (*red*), as a function of the target. As expected, muscles were more active during perturbed blocks with respect to baseline block.

Each muscle applies an end-point force along one direction, its ‘pulling vector’, i.e. the column of the ***H*** matrix corresponding to that muscle. The direction of the pulling vector generally does not coincide with the direction of peak activation [40]. During isometric conditions, if no co-contraction is required and the target force has a direction that is opposed to the pulling direction of a muscle, that muscle should not be active [38,41]. Data recorded during the baseline block (target directions 2, 13, and 18 for BicShort, 7 and 19 for TriLong, 5, 7, and 19 for DeltM in the examples of Fig 8, *blue lines*) indeed showed that most muscles exhibited little activation for targets in directions opposite to their pulling vector. In contrast, data recorded during perturbed blocks show that most muscles were active for all target directions. This indicates that, if the task requires stiffening the arm while exerting an isometric force along a direction, the CNS activates muscles that were silent during the pure force exertion task, possibly to counteract the forces generated by other muscles whose activity increases to module co-contraction. To quantify the increase in muscle activation we then computed the norm of the muscle activation vector. Significant effects on the norm of the muscle activation vectors were observed for the block factor (F_1,1778_ = 696.3, p < 0.001). The Welch t-test comparisons indicated that significant differences were present between baseline block and the other perturbation conditions and between B4 and both B5 and B6 but not between B5 and B6 (B2-B4: t(699.76) = -22.9, p < 0.001; B2-B5: t(741.3) = -28.3, p < 0.001; B2-B6: t(761.46) = -30.4, p < 0.001; B4-B5: t(891.78) = -2.4, p = 0.02; B4-B6: t(905.02) = -4.1, p < 0.001; B5-B6: t(913.88) = 1.86, p = 0.06).

**Fig 9:**
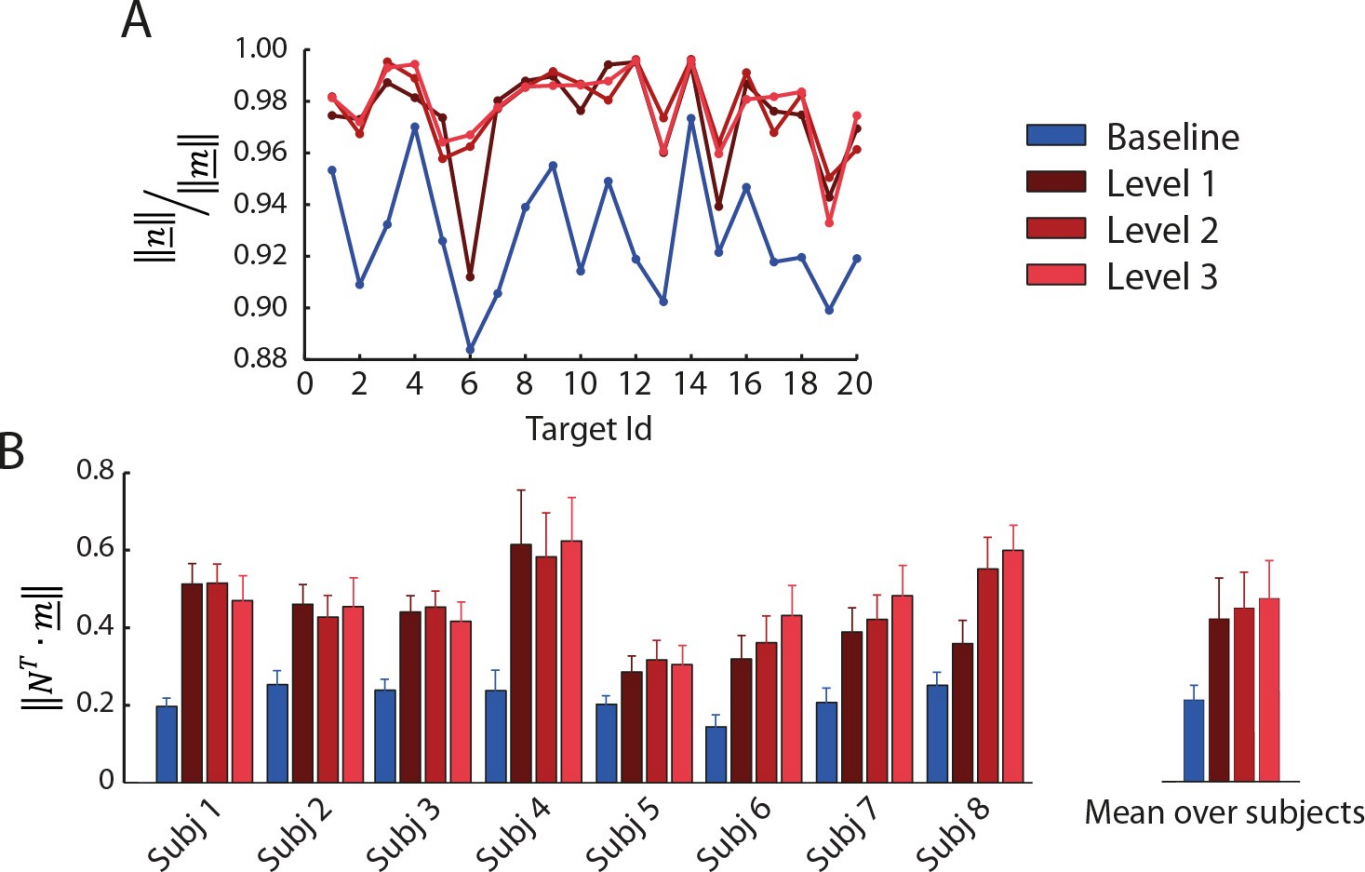
Null space component of the muscle activation vector. (**A**) Example of the ratio between the norm of the muscle activation vector and the norm of the projection of the muscle activation vector in the null space of the ***H*** matrix. Data were recorded from subject 8 in the baseline block (*blue*) and in perturbed blocks with magnitude level 1 (*dark red*), level 2 (*medium red*) and level 3 (*light red*) and averaged over repetitions of the same target. (**B**) Null space projection of the muscle activations, normalized to MVC, of successful trials averaged during hold phase and repetitions (mean ± SD among directions and repetitions) for all subjects. On the right side, the null space projection of the muscle activations, normalized to MVC, of successful trials averaged during hold phase and repetitions was averaged among subjects (mean ± SD across subjects).

To assess how the magnitude of the null space projection with respect to the magnitude of the muscle activation vector changes with co-contraction, we computed their ratio for each target direction (Fig 9A). As expected, the norm of the null space component in perturbed blocks was higher than in the baseline block. The mean fraction observed in the baseline block was: 0.909 ± 0.021 (mean ± SD across subjects of the fraction averaged for each subject across directions), and was statistically different from the mean fraction observed in the perturbed blocks (0.966 ± 0.010 in the perturbed block with perturbation magnitude level 1, 0.970 ± 0.012 in perturbed block with level 2, 0.968 ± 0.013 in perturbed block with level 3). The statistical difference was tested with a two-sided Wilcoxon rank sum test with a p-value threshold equal to 0.05.

Even in the baseline condition, the null space projection represented a large fraction of the norm of the muscle activation vector. As a non-zero projection of the muscle activation vector in the null space is necessary to satisfy the physiological constraint of non-negativity of the muscle activations, we wondered whether the observed fraction of the norm of the null space component could be due to the non-negativity constraint rather than to a neural control strategy. Thus, we estimated the non-negative muscle activation vector with minimum norm required to generate the applied force (see Methods) and projected such vector onto the null space. The average ratio between the norm of this projection and the norm of the baseline muscle activation vector across directions and subjects was 0.60 ± 0.06 (mean among subjects ± SD), which was tested to be significantly different from the ratio observed in all blocks and all subjects with a Wilcoxon rank sum (p-value threshold 0.05) and smaller than it. This indicated that a component of the observed muscle activation vectors was not due to the constraint of non-negative muscle activation.

Fig 9B shows the norm of the projection of the muscle activation vector in the null space (mean ± SD across directions) for all subjects and all blocks. Significant effects on the norm of the null space projection of the muscle activation vectors were observed for the block factor (F_1,1778_ = 713.3, p < 0.001). The Welch t-test comparisons indicated that significant differences were present between baseline block and perturbed blocks and between the perturbed blocks (B2-B4: t(708.84) = -23.5, p < 0.001; B2-B5: t(748.57) = -28.8, p < 0.001; B2-B6: t(773.18) = -30.8, p < 0.001; B4-B5: t(892.83) = -2.43, p = 0.015; B4-B6: t(903.68) = -3.94, p <0.001; B5-B6: t(913.53) = 1.6, p = 0.11). Thus, subjects increased the null space component of their muscle activation vector as the level of the perturbing force increased.

### Cosine tuning

**Fig 10:**
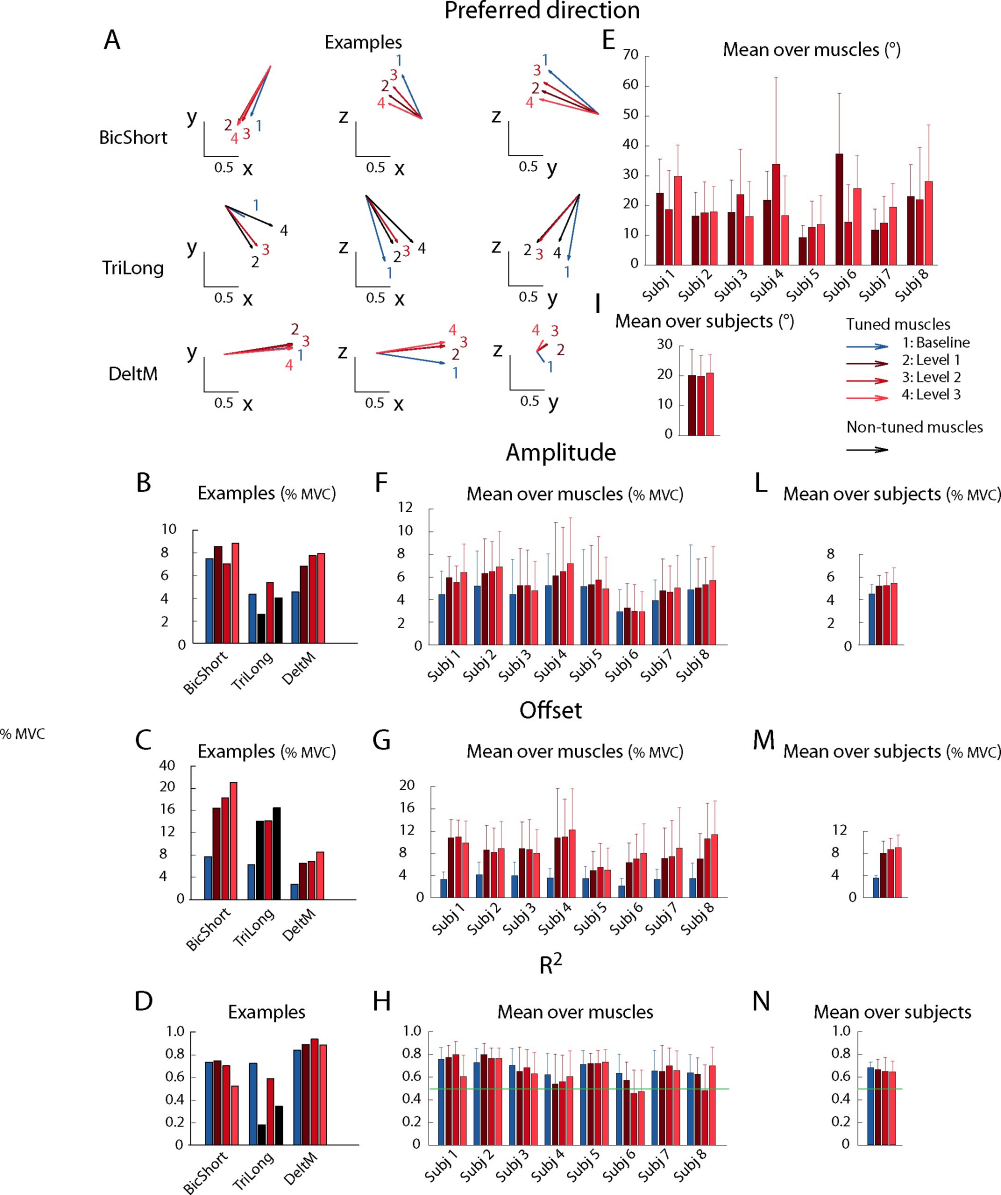
Cosine tuning of muscle activation. A-D: Example of the parameters of a spatial cosine function characterizing the directional tuning of three muscles (Biceps short head, Triceps long head, and Deltoid middle) of subject 8. Muscles with non-significant fit (p ≥ 0.05) or low quality of the fit (R^2^ ≤ 0.5) for each noise level is reported in black, otherwise baseline (no noise) is reported in blue and perturbed blocks in red (perturbation magnitude level 1 in *dark red*, level 2 in *medium red* and level 3 in *light red*). Cosine tuning functions are fitted on MVC normalized muscle activations and therefore amplitude and offset also represent normalized values. (**A**) Projection of the preferred directions on the horizontal (x-y, first column), frontal (x-z, second column) and sagittal (y-z, third column) planes (the preferred directions amplitude was normalized to 1). (**B**) Amplitude of the cosine tuning (data normalized to MVC). (**C**) Offset of the cosine tuning. (**D**) R^2^ of the reconstruction. E-H: Comparison of the cosine tuning for different blocks (mean ± SD) among muscles with significant (p < 0.05) and good quality (R^2^ > 0.5) fit in the baseline block and in at least one perturbed blocks. (**E**) Angle (in degrees) between the preferred directions in the baseline block and in perturbed blocks. (**F**) Amplitude of the cosine tuning. (**G**) Offset of the cosine tuning. (**H**) R^2^ of the cosine fit. I-N: Comparison of the cosine tuning for different blocks, mean ± SD among subjects. (**I**) Angle (in degrees) between the preferred direction in the baseline block and in the perturbed blocks. (**L**) Amplitude of the cosine tuning. (**M**) Offset of the cosine tuning. (**N**) R^2^ of the cosine fit.

The dependence on the target of the activation of most muscles could be fitted by a spatial cosine function (Fig 10). The number of muscles with a non-significant (i.e., p ≥ 0.05) cosine fit was 1.1 ± 0.8 (mean ± SD across subjects, n = 8) in the baseline block, 1.6 ± 2.1 in the perturbed block with perturbation magnitude level 1, 2.5 ± 3.4 in the perturbed block with level 2, and 2.1 ± 2.5 in the perturbed block with level 3. Thus, most of the 17 recorded muscles had a significant cosine-tuning in all conditions even if there was a small decrease in the number of muscles with a significant cosine-tuning when higher co-contraction was required. As muscle activation could have a significant cosine tuning even if the cosine function did not capture most of the variation, we also quantified the number of muscles with a R^2^ value lower than 0.5. The number of muscles with a low quality of the cosine tuning fit (R^2^ ≤ 0.5) was 2.2 ± 1.6 (mean ± SD across subjects, n = 8) in the baseline block, 3.5 ± 3.3 in the perturbed block with perturbation magnitude level 1, 4.0 ± 4.3 in the perturbed block with level 2, and 3.4 ± 3.1 in the perturbed block with level 3. We then selected for further analysis only the activations of the muscles with a significant cosine tuning and a value of the cosine fit R^2^ > 0.5. The number of muscles whose baseline block activation and at least one perturbed block activation satisfied these selection criteria was 14.1 ± 2.3 (mean ± SD across subjects, n = 8) out of 17 recorded muscles. Thus, most of the muscles, in each subject, had activations that were cosine tuned both in the baseline block and in at least one perturbed block. The number of perturbed blocks in which muscle activation was cosine-tuned, if the baseline block activation was cosine-tuned, was 2.7 ± 0.7 out of 3 perturbed blocks. Thus, a muscle that was cosine-tuned in the baseline block was in most cases also cosine-tuned in at least two of the perturbed blocks.

The angles between the preferred directions (Fig 10E) in baseline and perturbed blocks were calculated together with the cosine tuning amplitude (Fig 10F), offset (Fig 10G) and R^2^ (Fig 10H) values. Examples of the cosine tuning parameters for three muscles (as in Fig 8) are shown in Fig 10A, 10B, 10C and 10D. The mean angle between the baseline preferred direction and the preferred direction in any perturbed block, for muscles with a significant and high R^2^ (> 0.5) cosine tuning, was 21.2° ± 5.6° (mean ± SD across subjects, Fig 9E). The percent increase in the values of the cosine tuning amplitude and offset in any perturbed block with respect to the baseline block were 38% ± 13% (mean ± SD across subjects, Fig 9F) and 166% ± 73% (mean ± SD across subjects, Fig 9F) respectively. Thus, when increased co-contraction was required, the preferred direction slightly rotated while the amplitude and especially the offset of the cosine tuning increased. Statistical analysis revealed significant effects of the block factor on the cosine tuning amplitude (F_1,436_ = 10.18, p < 0.01) and offset (F_1,436_ = 87.32, p < 0.001). Post-hoc comparisons (Welch test) identified significant differences between the baseline block and perturbed blocks but not between the perturbed blocks both for the cosine tuning amplitude (B2-B4: t(236.56) = -2.1, p = 0.04; B2-B5: t(215.35) = -2.6, p = 0.01; B2-B6: t(219.27) = -2.9, p <0.01; B4-B5: t(209.8) = -0.47, p = 0.63; B4-B6: t(214.8) = -0.9, p = 0.37; B5-B6: t(211) = 0.44, p = 0.66) and offset (B2-B4: t(149.2) = -8.5, p < 0.001; B2-B5: t(130.15) = -8.7, p < 0.001; B2-B6: t(137.54) = -9.24, p < 0.001; B4-B5: t(199.65) = -1.32, p = 0.19; B4-B6: t(207.97) = -1.7, p = 0.1; B5-B6: t(210.58) = 0.29, p = 0.77). Furthermore, there was a significant effect of block pair on the cosines of the angles between preferred directions (KW: df = 5, χ^2^ = 16.84, p < 0.01). Post-hoc tests however showed that only the difference between the cosines of the angles between the preferred directions in the baseline (B2) and in the last perturbed blocks (B6) and between the first (B4) and in the second (B5) perturbed blocks was significant (p = 0.016).

### Null space components

As muscle activation vectors generating the same force with different levels of co-contraction differ only in their projection onto the null space of the EMG-to-force matrix, to characterize the co-contraction strategies we first tested whether subjects increased their co-contraction by linearly scaling the null space projection observed in the baseline condition (hypothesis 1). Such scaling strategy predicts, in the absence of any physiological variability in the activation of the muscles and EMG noise, the cosine of the angles between the null space component in the baseline block and those in the perturbed blocks to be equal to 1. To assess the significance of a deviation from a cosine value of 1 in presence of variability of the EMG signal, we estimated the distribution of the values of the cosine of the angles between the null space projections recorded during different repetitions of the same baseline target. The distribution of the cosines of the angles between the null space projection recorded during the block with disturbance magnitude level 1 and the null space projections recorded during the baseline block was significantly different (p < 0.05) from the distribution of the cosines of the angles among the different repetitions of the baseline block in 5 out of 8 subjects (subject id: 1, 2, 3, 4, 7; p-values of subjects 5, 6, 8 were respectively: 0.203, 0.233, and 0.488). In the block with disturbance magnitude level 2 the differences were significant in 6 out of 8 subjects (subject id: 1, 2, 3, 4, 5, 7; p-values of subjects 6, 8 were respectively: 0.094, and 0.054) and in the block with disturbance magnitude level the differences were significant in all subjects. Therefore, as a non-collinearity between the null space projection of the muscle activation recorded during the perturbed blocks and the null space projection of the muscle activation recorded during the baseline was observed in most subjects and disturbance magnitudes, the first hypothesis was not supported.

We then tested whether the changes in null space components observed when higher co-contraction was required could be explained by scaling of the null space vector representing the difference between the observed muscle activation vector (**m**_**i**_, i = 2 for baseline, i = 4,5,6 for perturbed blocks) and the non-negative minimum-norm muscle activation vector (**m**_**0**_ see Methods) for each given target (hypothesis 2). The distribution of the cosine of the angles between the **m**_**4**_-**m**_**0**_ recorded during the block with disturbance magnitude level 1 respect with the **m**_**2**_-**m**_**0**_ recorded during the baseline block was significantly different (p < 0.05) from the distribution of the cosines of the angles among the different repetitions of the baseline block in 5 out of 8 subjects (subject id: 1, 2, 3, 4, 7, p-values of subjects 5, 6, 8 were respectively: 0.186, 0.122, and 0.723). In the case of disturbance magnitude level 2 the difference was significant in 6 out of 8 subjects (subject id: 1, 2, 3, 4, 5, 7, p-values of subjects 6, 8 were respectively: 0.073, and 0.365) and in the case of disturbance magnitude level 3 the difference was significant in all subjects. Thus, in most cases **m**_**4,5,6**_-**m**_**0**_ recorded during perturbed blocks was not obtained by scaling the **m**_**2**_-**m**_**0**_ recorded during the baseline block, indicating that also the second hypothesis was not supported.

We then further tested whether the changes in null space components observed when higher co-contraction was required could be explained by scaling of the null space vector representing the difference between the muscle activation vector recorded during the perturbed blocks (**m**_**4, 5, 6**_) and the muscle activation vector recorded during the baseline block (${\bar{m}}_{2}$) for each given target (hypothesis 3). The muscle activation recorded during the baseline block, averaged among the repetitions of the same target, was subtracted from the muscle activation recorded during each repetition of perturbed blocks ($m_{i}-{\bar{m}}_{2}$). The distribution of the cosine of the angles among all the pairs of $m_{i}-{\bar{m}}_{2}$ recorded during blocks with different disturbance magnitude levels resulted to be significantly different (p < 0.05) from the distribution of the angles among the different repetitions of the $m_{i}-{\bar{m}}_{2}$ calculated during the baseline block in all subjects and disturbance magnitude levels. Thus, also the third hypothesis on a scaling rule was not supported as the null space projections of $m_{4,5,6}-{\bar{m}}_{2}$ among different perturbed blocks were not collinear.

Finally, as none of three scaling hypotheses was supported, we tested whether the modulation of the null space projections during perturbed block could be obtained as a linear combination of the null space projections observed for each target in the baseline condition, in which only the generation of force was required, and the null space vector generated in the pure co-contraction condition (**m**_**3**_), in which subjects had to voluntarily increase co-contraction without generating force (hypothesis 4). As the cosine of the angle between **m**_**3**_ and its null space projection was 0.98 ± 0.03 (mean ± SD across subjects), we verified, as expected, that it belonged to the null space. The distribution of the angle between **n**_**i**_ (i = 4, 5, 6) averaged on repetitions of the same target, and the subspace spanned by **n**_**2**_, averaged on repetitions of the same target, and **m**_**3**_, also averaged on repetitions, was compared with the distribution of the angles between different repetitions of the **n**_**2**_ and the same subspace due to noise (see Methods). The distribution of the angles between **n**_**i**_ the subspace spanned by **n**_**2**_ and **m**_**3**_, was significantly higher than the distribution of the angles between different repetitions of **n**_**2**_ and the same subspace in all perturbed blocks of only one subject (subject 1, p = 0.015 for block 5, p < 0.001 for blocks 5 and 6) and in only one perturbation magnitude level of a second subject (block 4 of subject 4, p < 0.001). Therefore, in most subjects and disturbance magnitudes, the null space projection of the muscle vector generated during a combined force and co-contraction task was achieved by a linear combination of the null space projection of muscle activation recorded during a force-only task and the null space vector recorded during a pure co-contraction task.

## Discussion

We investigated muscle coordination underlying voluntary modulation of co-contraction during the generation of isometric forces in different spatial directions. Many unstable manipulation tasks involved in everyday activities require the stiffness regulation by modulating co-contraction while generating end-point forces. We aimed at characterizing co-contraction during force generation in multiple directions over 17 muscles including most of the muscles that generate force at the hand. We used a virtual manipulation environment with a visual perturbation to induce systematic changes of endpoint force direction and muscle co-contraction. Subjects had to displace a cursor in space and reach one of 20 targets by applying isometric forces at the hand. When a sinusoidal perturbation was applied to the cursor, to keep the cursor within the target subjects were asked to reduce the magnitude of the oscillation of the cursor by co-contracting their arm muscles. All subjects were able to reach the target and to maintain the cursor within the target in at least one trial at each of three perturbation magnitude levels and, on average, in at least two thirds of the trials. Subjects succeeded by both increasing co-contraction, thus reducing the magnitude of the cursor oscillation, and by generating forces that matched the target more accurately, thus letting the cursor oscillate around a point closer to the center of the target sphere and thus better exploiting the target tolerance. In terms of individual muscles, in most cases the directional modulation of activation was well captured by a spatial cosine function both during baseline and when the perturbation was applied. When a higher level of co-contraction was required, the cosine function’s offset increased largely and its amplitude slightly. With regards to muscle patterns, higher co-contraction was associated to an increase in magnitude of the null space projections. However, even in the baseline condition, null space magnitude was higher than the minimum possible magnitude, estimated for each force target as the null space projection of the minimum-norm non-negative muscle pattern. Moreover, the increase in magnitude of the null space projection was not achieved following one of three tested vector or affine scaling rules. Neither the null space vector observed in the baseline condition, nor the difference between the baseline null space vector and the minimum-norm non-negative null space vector, nor the difference between the perturbed blocks null space vector with the smallest disturbance magnitude and the baseline null space vector were scaled when increased co-contraction was required. However, the null space projections of the muscle vectors observed during the combined force and co-contraction task, were achieved as a combination of the null space projections of the muscle vectors observed during the generation of only force and the muscle vectors generated for a pure co-contraction task.

### A novel approach to study voluntary modulation of co-contraction

Co-contraction modulation has been investigated before but mostly in a few muscles. Co-contraction in muscles with opposite mechanical actions around some joint increases in tasks that require the stabilization of the position of that joint under mechanical perturbations or destabilizing forces. Hogan [1] recorded surface EMG from a pair of forearm flexor and extensor muscles while subjects maintained the upper arm horizontal and the forearm flexed in unstable vertical orientation and held a load in the hand. De Serres and Milner [5] showed that co-contraction of three wrist flexors and one wrist extensors increased when wrist angle had to be maintained while operating against an unstable load. Lacquaniti and collaborators [12,42] showed that hand impedance is modified when catching a falling ball through both anticipatory and reflexive co-contraction, with a transient reversal of the direction of stretch reflex responses centered on impact. Modulation of co-contraction has also been observed for the adaptation of reaching movements under perturbing forces. Thoroughman and Shadmehr [13] recorded EMG from four muscles as subjects learned to move a manipulandum that created systematic velocity-dependent forces. Co-contraction in two pairs of antagonist muscles was high when subjects were initially exposed to the force field and then rapidly declined during learning. Burdet and collaborators [8] showed that with practice subjects learned to make straight movements in a destabilizing divergent force field by selectively increasing stiffness in the direction of instability. While early in the learning period there was an increase in agonist-antagonist co-contraction [15], as the subject became more successful in counteracting the instability of the divergent field, the EMG was gradually reduced but remained higher than for reaching without perturbation [43]. While co-contraction is automatically regulated during unstable interactions with the environment, it can also be voluntarily increased. Gomi and Osu [28] studied the controllability and spatial characteristics of hand impedance during different co-contractions while maintaining a given arm posture. Subjects received visual feedback on the EMG activation level of three pairs of antagonist muscles as bar graphs on a monitor and were instructed to match specific activation levels indicated by reference markers, thus allowing to voluntary regulate co-contraction either in all muscle pairs or at specific monoarticular muscle pairs. Even if subjects could modulate co-contraction and regulate the joint stiffness ratio at the elbow and shoulder joint during posture maintenance, the change of stiffness geometry was limited. Perreault and collaborators [19] also found that subjects were able to voluntarily change stiffness orientation when provided with real-time visual feedback of end-point stiffness but the magnitude of these changes was small. Recently, the capability of human subjects to decouple stiffness and force control by voluntary co-contraction has been investigated during thumb-index finger grip [44]. Subjects could decouple grip stiffness from force when using co-contraction on average by about 20% of the maximum measured stiffness over all force levels. In sum, several previous studies have demonstrated the capability of modulating co-contraction in a few pairs of antagonistic muscles either for stabilizing posture and movement or voluntarily through visual feedback of EMG activity or estimated stiffness.

Our goal was to characterize co-contraction in most of the shoulder and arm muscles generating force at the hand, and to relate their activity modulation across force directions to their mechanical action. Using isometric force generation, we could adequately characterize the endpoint force generated by each muscle using a linear mapping, which could be estimated by multiple linear regression. However, as there was no mechanical instability at the hand, rather than instructing subjects to modulate co-contraction by providing feedback of the activity of individual muscles, which would have been a highly non-intuitive and challenging task with many muscles [28,29], we simulated a controllable perturbation in a virtual environment [33]. Subjects received feedback of the generated isometric force spatial vector as the displacement of a spherical cursor from a rest position matching the center of their palm displayed stereoscopically on a mirror occluding their hand and forearm [32,34,45]. Generation of a specific force target was thus achieved as reaching a corresponding spatial target and it was then possible to simulate instability by perturbing the cursor. Because the cursor position was simulated in real-time as the position of a mass coupled through a spring to the position of a second mass coupled through a second spring to the center of the palm, subjects controlled the mean position of the cursor by generating isometric force on the first mass and the amplitude of the cursor oscillation under a perturbing sinusoidal force by setting the stiffness of the second spring with their muscle activation. The magnitude of the stiffness was regulated according to the magnitude of the projection of the instantaneous muscle activation vector onto the null space of the linear mapping of muscle vectors into endpoint force, i.e. the space of all muscle patterns not affecting endpoint force. Such null space thus represents a multidimensional generalization of the notion of co-contraction of a pair of agonist-antagonist muscles. Importantly, as our aim was to investigate muscle coordination during co-contraction, with this approach we did not bias the selection of a specific co-contraction pattern as we would have done by instructing subjects to achieve a specific level of activation in each muscle with muscle-specific visual feedback. While subjects could have selected any null space vector to increase the stiffness of the virtual spring and to reduce the oscillation of the cursor, it is reasonable to assume that they used a strategy normally employed to reject a perturbation applied at the hand during a quasi-static manipulation task that requires generating a force in a specific direction. Thus, we could systematically investigate voluntary modulation of co-contraction in an intuitive and easy to perform task with a well-defined mechanical characterization of muscle action that reproduced some of the features of the natural control of impedance during unstable manipulation. Finally, as the present approach relies on the characterization of co-contraction during isometric force generation, it allows to investigate the feed-forward control strategies used to deal with disturbances independently of the contribution from reflexes that would be active with real perturbations.

### Modulation of activity in individual and multiple muscles

We first characterized co-contraction at the level of individual muscles. In accordance with previous studies [32,37,39] we found that in baseline conditions, i.e. when generating isometric forces in different directions without increased co-contraction, muscle activation was modulated by force direction and the directional tuning could be approximated, in most cases, by a cosine function. We thus wondered how co-contraction affected the directional tuning. We then characterized the changes in the parameters describing a spatial cosine function: preferred direction, amplitude and offset. We found small changes in the preferred direction and large changes in amplitude and especially in offset. Such changes are compatible with a global control of co-contraction, i.e. a control strategy that affects muscle activation over multiple target directions, thus modulating the parameters of the entire directional tuning curve. In contrast, since each muscle has a preferred direction along which its activation is maximal, a local control of co-contraction might affect only the activation of those muscles whose preferred direction is either aligned with the target direction or in the opposite direction, i.e. those muscles that most closely approximate at the endpoint the notion of agonist and antagonist muscles around a single joint. However, according to such a control strategy, different muscles would be modulated for different target directions and, then, co-contraction would mostly distort rather than modulate the cosine tuning. As most of the muscles had a significant (p < 0.05) and good quality (R^2^ > 0.5) cosine tuning both in baseline and in perturbed conditions, while we cannot rule out a contribution of a local control strategy, the data suggest that the control of co-contraction occurs mostly by an increase in activation of each muscles across all directions according to a direction-independent amount (cosine offset) with some contribution from a directional-dependent component (cosine amplitude).

We then characterized co-contraction in terms of coordination of multiple muscles by projecting the observed muscle activation vectors onto the null space of the EMG-to-force mapping. Surprisingly, we found that, even in the baseline, the magnitude of the null space projection was on average across subjects and target direction over 90% of the magnitude of the entire muscle activation vector (Fig 9A). As the dimensionality of the null space (14 for 17 recorded muscles) is much larger than the dimension of the orthogonal force space (3), we wondered whether such a large magnitude of the null space projection was an obligatory consequence of the fact that physiological muscle patterns are represented by non-negative vectors. Indeed, mathematically, to achieve a specific force with a non-negative muscle vector it is necessary to combine an appropriate null space vector to the minimum-norm muscle activation vector in the force space, which in general is not a non-negative vector. We then compared the norm of the null space projections of the muscle vectors observed in the baseline condition across force targets with the norm of the null space projection of the minimum-norm, non-negative muscle activation vectors generating the same forces. We used quadratic optimization and subject-specific EMG-to-force mappings to estimate such minimum-norm vectors. The null space projections of the minimum-norm, non-negative muscle activation vectors had a smaller magnitude than the projections of the observed muscle activation vectors (66.5%), indicating that the level of co-contraction observed in the baseline was higher than the level imposed by the non-negativity constraints. This discrepancy could be ascribed to the higher co-contraction that might be required to maintain a specific posture in the setup. However, as the subject’s hand and forearm were fully supported and immobilized in a splint, no muscular activity was required to maintain a stable posture in the setup. High levels of co-contraction have been reported in the early stages of motor learning [2,13,15,46,47]. However, similar magnitudes of the null space projections were identified during the first and the last trial of the baseline block, so it is unlikely that this large co-contraction was a consequence of learning of a new task. The large magnitude of the null space projection may be due to a suboptimal recruitment of the muscles by CNS. Suboptimality might result from activating muscles within synergies rather than individually [32], in an habitual pattern [48], or according to a local rather than global optimization [49].

The magnitude of the projection of the muscle activation vector onto the null space further increased when additional co-contraction was required to attenuate the effect of the sinusoidal perturbation on the cursor (Fig 9A and 9B). As the task only required an increase of the magnitude of the null space projection, changes in the direction of the null space component may provide additional information on the coordination strategies used to modulate co-contraction. One simple strategy is to scale the null space component of the muscle activation vector selected in the baseline according the required level of co-contraction. As scaling of the entire muscle vector would increase force together with co-contraction, an independent modulation of the null space component is required. Scaling of the null space component observed in the baseline would result in collinear null space components during the perturbation. Alternatively, as we found that the baseline null space component was already larger than the minimum-norm non-negative null space component, the null space components in perturbed conditions might have resulted from scaling the increment observed in the baseline with respect to the minimum-norm non-negative null space component. Otherwise, since the minimum-norm non-negative solution may not be actually achieved by the CNS, the null space components in perturbed conditions might have resulted from scaling the increment observed in the perturbed condition with the smallest disturbance magnitude with respect to the baseline null space component. None of these three possibilities were supported by the data as the values of the cosine of the angles between the different null space vectors were significantly smaller that the values expected from EMG variability estimated by computing the cosine of the angles between different repetitions of the baseline trials to the same target for most participants and disturbance magnitude levels.

We then wondered whether the null space projection of the muscle activations, recorded during the three conditions in which subjects were asked to exert isometric force while increasing co-contraction, could be achieved by a combination of the null space components used during the generation of only force (baseline condition) and the muscle activation vector used to generate co-contraction without force (pure co-contraction condition). For most subjects and disturbance magnitude levels (20 out of 24) the null space projection of the muscle activation vector for each target lay on the subspace spanned by the null space projection of the baseline muscle activation vector and the pure co-contraction muscle activation vector, indicating that the modulation of the null space vector with co-contraction, rather than following a scaling rule, was achieved by the combination of the specific null space vector used to generate pure co-contraction to the baseline force-only vector used for each target. The existence of such linear combination rule suggests that subjects relied on a subject-specific co-contraction strategy (as the pure co-contraction muscle activation vectors differed across subjects, data not shown), that was used when an increase in co-contraction was required both with and without force generation. However, the pure co-contraction vector was not simply scaled with disturbance magnitude level, as in such a case the differences between the resulting null space vectors would have been aligned, and this possibility was tested for hypothesis 3.

Lack of scaling of the null space projection of the muscle activation vector or the null space component representing the difference between observed baseline muscle activation vector and minimum-norm non-negative solution or the null space component representing the difference between observed perturbed blocks muscle activation vector and baseline muscle activation vector indicates that the CNS selects a specific co-contraction pattern according to constraints or control strategies yet to be identified. Scaling might be a convenient control strategy if the CNS were free to choose any muscle activation vector in the null space, thus reducing the problem of selecting the appropriate null space vector by mapping a desired level of co-contraction onto the magnitude of a coefficient scaling the null space vector used in baseline conditions. However, the generation of null space vectors might be constrained, and it might not be possible to scale the null space component used in the baseline. As for the selection of force-generating muscle patterns [32,34,45,50], the CNS might rely on a small number of muscle synergies also to generate the appropriate co-contraction patterns. If so, the generation of null space vectors might be achieved by combining co-contraction specific muscle synergies, i.e. synergies which do not generate any force, or by specific combinations of force-generating synergies with zero resultant force. In both cases, the null space vector that must be added to the baseline null space vector to increase co-contraction must lie on the subspace generated by synergy combinations thus making a scaling strategy unfeasible if the null space vector to be scaled does not lie on the synergy subspace. Moreover, the selection of the co-contraction subspace of the null space might take into account the stiffness generated by co-contraction patterns. In fact, approximating the mapping of muscle activation vectors into stiffness ellipses as a linear mapping [31], one can define a null space of the muscle-to-stiffness matrix which is in general different from the null space of the muscle-to-force matrix. Thus, there are co-contraction patterns that are effective in modulating stiffness and co-contraction patterns that are not (i.e. those belonging to the intersection of the two null spaces). The CNS might organize synergies to simplify the generation of stiffness-modulating co-contraction patterns, even if such strategy has a cost in terms of effort and controllability of the orientation of the stiffness ellipse [31]. We plan to investigate the synergistic organization of co-contraction patterns in future work.

### Simplifying assumptions

Our experimental method relies on some simplifying assumptions. The relation between the rectified EMG signals and the force exerted by the arm was approximated as linear, a critical step in the methodology because it allowed to compute in real-time the projection of the muscle activation in the null space. Previous studies also modeled the relation between the EMG signals and the end-point force as linear [29,40,51]. Such approximation is reasonable for the generation of isometric forces with a magnitude much smaller than the MVF [52], as in our case (20% MVF). However, the validity of the assumption was tested computing the reconstruction of the end-point force as the product of the matrix that mapped the EMG onto the end-point force for the EMG signal. The one subject that showed a relatively low end-point force reconstruction (R^2^ = 0.61) was excluded from the dataset. Since the reconstruction of the end-point force was satisfactory in the other subjects (R^2^ > 0.75), we considered this simplifying assumption to be adequate for our purpose of identifying the null space projection of muscle activation vectors.

An additional simplification was the simulation of isotropic end-point stiffness. Differently from the human arm, whose end-point stiffness is known to be non-isotropic [53], the stiffness of the spring controlling the cursor oscillation induced by co-contraction was the same in all directions. However, when generating isometric forces, the end-point stiffness ellipse can be controlled mostly in magnitude rather than in orientation [19]. Moreover, in our experiment the perturbation amplitude was the same in all the three spatial directions and therefore there was no need to reduce the oscillation selectively along one direction. Thus, using an isotropic virtual stiffness simplifies the implementation of its online control without introducing a significative limitation of the control capability of the participants with respect to the control of the real end-point stiffness.

Finally, to test the collinearity among the null space vectors observed during different blocks, we assumed that subjects used a consistent co-contraction strategy to generate muscle patterns in different trials to the same end-point force target in the baseline block. Violation of this assumption would broaden the distribution of the cosine of the angles among baseline repetitions used to assess significant differences of the cosine of the angles between different blocks. The violation of this hypothesis, for example if subjects used different strategies in different trials to modulate co-contraction while generating the same force target, could explain why a few subjects did not show a significant difference in angle between the null space vectors tested when assessing different scaling laws.

## Conclusions

We demonstrated that it is possible to investigate systematically voluntary modulation of arm muscle co-contraction during multi-directional force generation using an isometric reaching task in a virtual environment with a simulated disturbance. The fact that co-contraction is regulated by increasing the amplitude and the offset of the cosine tuning of individual muscles, suggests a global rather than local control strategy. However, the fact that the coordination of multiple muscles cannot be explained by simple scaling of null space vectors suggests that the CNS is subject to constraints or exploits rules, such as muscle synergy combinations, yet to be identified when voluntarily modulating co-contraction.

## Supporting information

S1 Appendix Myoelectric control of virtual stiffness. (DOCX)

S1 FigMyoelectric control of virtual stiffness.(A) Logistic law used to determine the stiffness of the second mass-spring-damper system. The stiffness was related to the ratio between the projection of the muscle activation ***m*** in the null space *N* of the EMG-to-force matrix and its mean value calculated during the hold phase of the non-perturbed baseline block. (B) On *x* axis was reported the ratio between the pulsation *ω* of the applied force and the natural pulsation ωn=Kmm. On *y* axis was reported the ratio $\left|G\left(\omega \right)\right|=\frac{1}{\sqrt{{\left[1-{\left(\frac{\omega }{\omega_{n}}\right)}^{2}\right]}^{2}+4\zeta^{2}{\left(\frac{\omega }{\omega_{n}}\right)}^{2}}}$, between the response of the system to a unitary force with a pulsation *ω* and the displacement if the same force was statically applied. ζ=Dm2m⋅Km was the ratio between the actual and the critical damping.(TIF)

S1 Dataset Matlab structure containing EMG, force, and target information for all selected and successful trials of each subject. (MAT)

S1 Text Description of the Matlab structure contained in S1 Dataset. (TXT)
