# Supplementary material for: Muscle patterns underlying voluntary modulation of co-contraction
Source: PLoS One. 2018 Oct 19;13(10):e0205911. doi: 10.1371/journal.pone.0205911 (PMC6195298; doi:10.1371/journal.pone.0205911)
Supplement: S1 Appendix — (DOCX) [file pone.0205911.s001.docx]

**S1 APPENDIX**

Since the relation between the muscle activation and muscle stiffness is approximately linear and endpoint stiffness is the resultant of the linear combination between the muscles stiffness [[4](#_ENREF_4)] a linear relation between the null space projection of the muscle activation (***n***) and the endpoint stiffness (*K_m_*) was assumed. Subjects were given a feedback of ***n*** by the amplitude of the oscillation of the cursor.

A logistic law (S2 Fig, panel A) between *K_m_* and ***n*** was selected because it ensured a smooth transition between a lower and an upper asymptote. The lower asymptote would avoid low values of the stiffness and the consequent resonance, while the upper asymptote would avoid numerical resonance due to discretization. The logistic curve was univocally defined by three couples of stiffness ($K_{1}$, $K_{2}$, $K_{3}$) and ***n*** ($n_{1}$, $n_{2}$, $n_{3}$) values. The lower stiffness value $K_{1}$was selected to be close to physiological values of arm stiffness ($K_{1}=1000\frac{N}{m^{2}}$) and the upper value was selected to be higher than $K_{1}$ ($K_{3}=9\cdot K_{1}=9000\frac{N}{m^{2}}$). $K_{2}$ was selected to be closer to $K_{3}$ than $K_{1}$ ($K_{2}=7500\frac{N}{m^{2}}$) to ensure higher increments of stiffness, and consequently higher reduction of the cursor oscillation, for higher arm stiffness. Since the MVF differs among subjects, the virtual stiffness *K_m_* was related to the normalized value of ***n***. The value of ***n*** was normalized to its mean, recorded during the baseline block to ensure that each subject found the same difficulty in obtaining the same value of *K_m_*. $K_{1}$ was related to the value of ***n*** recorded during the baseline block ($n_{1}=1$) and $K_{3}$ was related to the higher feasible value of ***n***. Pilot experiments showed that $n_{3}$ could be set to 10. $n_{2}$ was the mean between $n_{1}$ and $n_{3}$ ($n_{2}=5.5$).

The equation of the logistic curve was:

$$K_{m}=\frac{c_{K}}{{a_{K}\cdot e}^{-b_{K}\cdot\boldsymbol{n}}+1}$$

where $a_{K}$, $b_{K}$ and $c_{K}$ are the coefficients of the logistic law defined by: $b_{K}=-log\left( \frac{K_{1}\left( K_{3}-K_{2} \right)}{K_{3}\left( K_{2}-K_{1} \right)} \right)$, $c_{K}=\frac{K_{1}\cdot K_{2}\left( 1-e^{-b_{K}} \right)}{K_{1}-K_{2}\cdot e^{-b_{K}}}$, and $a_{K}=\left( \frac{c_{K}}{K_{2}}-1 \right)\cdot e^{b_{K}\left( \frac{n_{2}}{n_{2}-n_{1}} \right)}$.

The damping *D_m_* was set to reduce the amplitude of the oscillation (*D_m_* *=* 0.5 times the critical damping $2\sqrt{m\cdot K_{m}}$). Since the stiffness $K_{m}$ depended on ***n***, as previously described, also the damping *D_m_* depended on ***n***. This approximation was in agreement with [[17](#_ENREF_17)] that observed a simultaneous rotation of the major axes of stiffness and damping ellipses. The spherical cursor mass *m* was set to *m =* 0.2 g. The virtual force (***F_n_***) applied to displace the cursor was the combination of three sinusoidal forces acting along the three dimensions of the force. ***F_n_*** high frequency would led the oscillation of the cursor to be perceived by the subject as to be pseudo-random. The experiment requested a reduction of the oscillation of *m* with the increase of the stiffness, i.e. the increase of the undamped natural frequency. For this reason, all the frequencies of the perturbing force components needed to be lower than the minimum natural pulsation ${\omega_{n}}_{min}=\sqrt{\frac{K_{1}}{m}}$ (S2 Fig, panel B), so the maximum pulsation frequency was selected such that $\omega_{max}\leq0.7\cdot{\omega_{n}}_{min}$. The constant 0.7 with a 0.5 critical damping, assured a response that grew with the stiffness in a limited range (if the stiffness was equal to $K_{1}$ the response was 1.16 times the static displacement). The chosen frequencies of the noise force ***F_n_*** were: 38 Hz along the *x* component, 30 Hz along the *y* component and 46 Hz along the *z* component. All those frequencies were high enough to ensure that the subjects did not voluntary compensate the oscillation by an opposite end-point force. Since the experiment required the subjects to stiffen their arm at different levels, three different levels of amplitude of ***F_n_***, chosen as a result of pilot experiments, solicited the mass *m*. The lower level (level 1) of arm stiffening would require little more activation in the null space, respect to the non-perturbed baseline force reaching task, to maintain the cursor inside the target, so the selected perturbing force was 50 N. The higher level (level 3) of arm stiffening required a high and slightly fatiguing, but still feasible, muscle activation in the null space to maintain the cursor inside the target, so the selected perturbing force was 80 N. Since the three amplitude levels were equally spaced the middle force level was set to 65 N.
