# Supplementary figures and images for: Muscle patterns underlying voluntary modulation of co-contraction
Source: PLoS One. 2018 Oct 19;13(10):e0205911. doi: 10.1371/journal.pone.0205911 (PMC6195298; doi:10.1371/journal.pone.0205911)

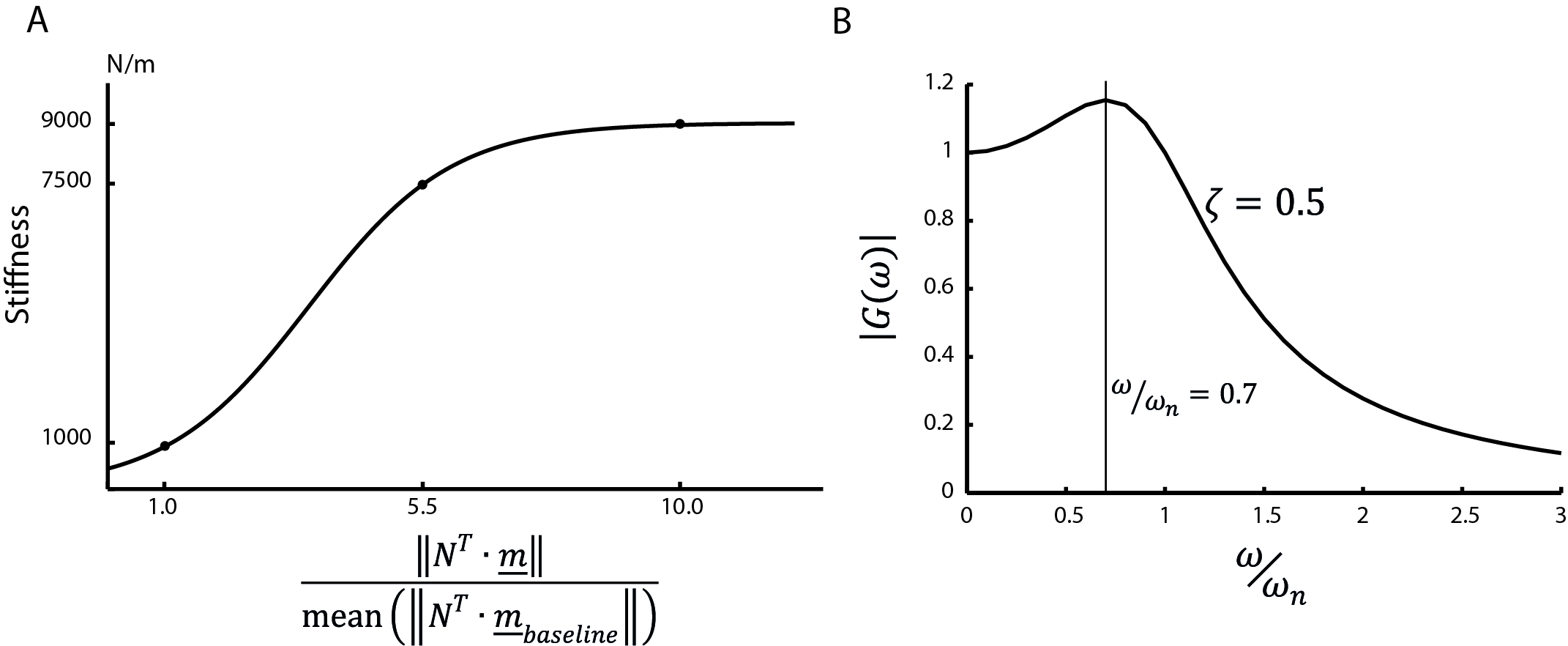

Supplement: S1 Fig — (A) Logistic law used to determine the stiffness of the second mass-spring-damper system. The stiffness was related to the ratio between the projection of the muscle activation m in the null space N of the EMG-to-force matrix and its mean value calculated during the hold phase of the non-perturbed baseline block. (B) On x axis was reported the ratio between the pulsation ω of the applied force and the natural pulsation ωn=Kmm. On y axis was reported the ratio |G(ω)|=1[1−(ωωn)2]2+4ζ2(ωωn)2, between the response of the system to a unitary force with a pulsation ω and the displacement if the same force was statically applied. ζ=Dm2m⋅Km was the ratio between the actual and the critical damping. (TIF) [file pone.0205911.s002.tif]
